# Anthocyanins, delphinidin-3-*O*-glucoside and cyanidin-3-*O*-glucoside, inhibit immune checkpoints in human colorectal cancer cells *in vitro* and *in silico*

**DOI:** 10.1038/s41598-019-47903-0

**Published:** 2019-08-09

**Authors:** Candice Mazewski, Morgan Sanha Kim, Elvira Gonzalez de Mejia

**Affiliations:** 1 Department of Food Science and Human Nutrition, University of Illinois, Urbana, USA; 2 Department of Chemical and Biomolecular Engineering, University of Illinois, Urbana, USA

**Keywords:** Computational chemistry, Cancer prevention, Diagnostic markers, Drug discovery

## Abstract

The objective was to assess anti-progression and stimulatory immune response effects among anthocyanins (ANC) and their metabolites on human colorectal cancer cells *in vitro* and *in silico*. Pure phenolics including delphinidin-3-*O*-glucoside (D3G) and its metabolites, delphinidin (DC) and gallic acid (GA), were tested alone or in combination, on HCT-116 and HT-29 human colorectal cancer cells (100–600 µg/mL). HCT-116 and HT-29 50% inhibition concentrations (µg/mL) were 396 ± 23 and 329 ± 17 for D3G; 242 ± 16 and >600 for DC; and 154 ± 5 and 81 ± 5 for GA, respectively. Using molecular docking, cyanidin-3-*O*-glucoside (C3G) showed the highest potential to inhibit immune checkpoints: programmed cell death protein-1 (PD-1) (−6.8 kcal/mol) and programmed death-ligand-1 (PD-L1) (−9.6 kcal/mol). C3G, D3G, DC, GA, and D3G-rich extracts decreased PD-L1 protein expression in HCT-116 cells. C3G decreased PD-L1 fluorescence intensity by 39%. ANC decreased PD-1 expression in peripheral blood mononuclear cells in monoculture by 41% and 55%, and co-culture with HCT-116 and HT-29 cells by 39% and 26% (C3G) and 50% and 51% (D3G), respectively. D3G and C3G, abundant in plant foods, showed potential for binding with and inhibiting immune checkpoints, PD-1 and PD-L1, which can activate immune response in the tumor microenvironment and induce cancer cell death.

## Introduction

In the United States, colorectal cancer deaths in 2018 were estimated at 50,630^1^. Currently, regional and distant stages represent 35% and 21% of the total colorectal cancer cases, and five-year survival rates are 71.1% and 13.8%, respectively, emphasizing the impact of metastasis on survival^1^. Three categories of drug therapies are used for metastatic colorectal cancer treatment: chemotherapy, targeted therapy, and immunotherapy. Chemotherapy drugs target highly proliferating cells while approved targeted therapies aim to inhibit proteins in the angiogenesis pathway, such as vascular endothelial growth factor (VEGF)^2^. More recently, VEGF has been shown, in addition to its proangiogenic effects, to hinder immune function in the tumor microenvironment by impacting infiltration of T cells^3,4^.

Immunotherapy drugs are the newest addition to combat colorectal cancer. In 2017, the first two immunotherapies (nivolumab and pembrolizumab) that target programmed cell death protein 1 (PD-1) were approved by the FDA for colorectal cancer with microsatellite instability-high or deficient mismatch repair solid tumors^5^. Microsatellites are short repeated sequences in the genome, and microsatellite instability indicates a disruption in the mismatch repair system and greatly increased mutations^6^. PD-1 is part of a group of immune checkpoints, which are negative regulators of the immune system and are targeted in immunotherapy to help activate T cell response against tumors^5^. Other immune checkpoints, including programmed death-ligand 1 (PD-L1), the ligand for PD-1, and cytotoxic T-lymphocyte-associated antigen 4 (CTLA-4), are also being targeted for treatment in colorectal cancer^7^. PD-L1 is typically overexpressed in tumor cells, contributing to the tumor cells’ ability to avoid immune destruction; when it binds to PD-1 on T cells, they are deactivated and unable to destroy the cancer cells^7^. In July of 2018, a combination treatment of nivolumab and ipilimumab, a CTLA-4 inhibitor, was approved by the FDA^8^. More successful therapies and the use of combination therapies are needed to enhance the highly complex mechanisms of the immune system in the tumor microenvironment^4,9^.

A diet high in non-starchy vegetables, fruits, and whole grains is recommended for decreasing the risk of developing colorectal cancer^10^. Anthocyanins (ANC) are phenolic compounds and natural pigments that provide blue, purple, or red color to fruits, vegetables, cereals, and legumes. ANC have been shown to have anti-carcinogenic properties including an impact on autophagy regulation in tumor development^11–14^. ANC metabolites, including phenolic acids, have been shown to reach the colon during *in vivo* studies on bioavailability^15^. Assessment of ANC and immune response related to carcinogenesis has not been well examined. ANC from black rice promoted an immune response by increasing T cell and macrophage populations in a leukemia *in vivo* model^16^. *In vitro* and *in silico* analyses of compounds present in ANC-rich extracts can provide more information on their mechanisms of action as it applies to the immune response in the tumor microenvironment.

Our previous studies with colorectal cancer and ANC-rich extracts have shown the potential for such extracts to inhibit colorectal cancer cell growth *in vitro*, with effects on markers of apoptosis, cell cycle, and angiogenesis^17,18^. However, in the present study we focused on pure ANC and their metabolites and extensively evaluated their effects on immune checkpoints. The objective of this research was to compare the anti-progression and stimulatory immune effects among ANC and their metabolites on human colorectal cancer cell lines, HCT-116 and HT-29. We employed a co-culture set-up in addition to the monoculture analyses to test ANC in a more representative model of the tumor microenvironment. Our findings support the potential for ANC to inhibit immune-suppressing markers, providing a basis for additional studies on the impact of other plant phenolics on immune response.

## Materials and Methods

### Materials

Human colorectal cancer cells, HCT 116 and HT-29, were purchased from the American Type Culture Collection (Manassas, Virginia) and human peripheral blood mononuclear cells (PBMC) from BioIVT (Hicksville, New York). The CellTiter 96^®^ AQueous One Solution Cell Proliferation Assay was purchased from Promega Corporation (Madison, WI) and the Pierce LDH Cytotoxicity Assay Kit from ThermoFisher Scientific (Waltham, MA). Delphinidin-3-*O*-glucoside (D3G), cyanidin-3-*O*-glucoside (C3G), malvidin-3-*O*-glucoside (M3G), delphinidin chloride (DC), procyanidin B1 (PB1), naringenin-7-*O*-glucoside, eriodictyol-7-*O*-glucoside, and quercetin-3-*O*-glucuronide (Q3G) all with >95% purity were purchased from ExtraSynthese (France). Red grape extract (RGE) was obtained from San Joaquin Valley Concentrates (Fresno, CA) and the black lentil extract (BLE) from the Kraft Heinz Company (Chicago, IL); both extracts were further purified as described in our previous publication^18^. Primary antibodies programmed cell death ligand-1 (1C10) (sc-293425), VEGF (F-5) (sc-365578), and GAPDH (sc-25778), and the radioimmunoprecipitation (RIPA) buffer were purchased from Santa Cruz Biotechnology (Santa Cruz, CA). Secondary antibody, anti-mouse IgG horseradish peroxidase conjugated, was obtained from GE Healthcare (Buckinghamshire, UK). Anti-PD-L1 antibody [EPR19759] (ab213524) was purchased from Abcam (Cambridge, UK). Atezolizumab and pembrolizumab were bought from BioVision, Inc. (Milpitas, CA). DC protein assay materials were obtained from Bio-Rad (Hercules, CA). All other chemicals used in this study were purchased from Sigma- Aldrich (St. Louis, MO) unless otherwise stated.

### Cell culture

HCT 116 and HT-29 cells were cultured in Minimum Essential Medium Eagle and PBMC in Roswell Park Memorial Institute (RPMI) medium both with 10% FBS, 1% penicillin/streptomycin, and 1% sodium pyruvate at 37 °C in 5% CO_2_ and 95% air. When HCT 116 cells were co-cultured with PBMC they were grown in RPMI medium. As microsatellite stability is important in the treatment of colorectal cancer, one cell line with a microsatellite instable phenotype (HCT 116) and another cell line with chromosomal instability (HT-29) were selected to allow for comparison of immune response effects^19^.

### HCT 116 and HT-29 cell viability with phenolics

The CellTiter 96® AQueous One Solution Cell Proliferation Assay was used to determine the most potent of the pure phenolics and the concentration for 50% inhibition (IC_50_) values for the ANC and metabolites on the HCT 116 cells based on the method described in our previous publication^17^. Briefly, cells were seeded in a 96 well plate at 1 × 10^4^ cells per well for 24 h. Pure phenolics alone or in combinations were added up to 600 µg/mL (50, 100, 200, 400, and 600 µg/mL) and incubated with the cells for 24 h. Ratios (percentages) for the phenolic combinations were based on the three main phenolics discovered in three extracts previously found to be potent at colorectal cancer cell inhibition: D3G, M3G, and Q3G (57:33:10) representing red grape; PB1, D3G, and Q3G (42:40:18) for black lentil; and PB1, eriodictyol 7-*O*-glucoside, and naringenin 7-*O*-glucoside (40:34:26) for sorghum^18^. Because cyanidin is the most ubiquitous anthocyanidin in fruits and vegetables and C3G was present in at least small amounts in all of the extracts tested in our previous study, it was tested individually, as well^20^. Some of the phenolics tested in the combinations are not water-soluble (Q3G, eriodictyol 7-*O*-glucoside, naringenin 7-*O*-glucoside) and had to be dissolved in DMSO, so they were not tested individually. A maximum of 0.5% DMSO was used on the cells, and an untreated control at the same percentage was always used for comparison. Additionally, two metabolites of D3G were tested: DC and gallic acid (GA). These were identified as metabolites based on the literature^21,22^. Absorbance using the CellTiter assay was calculated and compared at 515 nm with an ELX808IU Ultra Microplate Reader (BioTek, Winooski, VT). Then, IC_50_ values were calculated using Prism 6 software (GraphPad Software, La Jolla, CA).

### Interaction analysis of delphinidin-3-*O*-glucoside and anthocyanin-rich extracts with oxaliplatin

Oxaliplatin is a commonly used chemotherapy drug for colorectal cancer, so it is important to learn about potential chemical interactions. Isobolograms were utilized to analyze the effect of ANC-rich extracts, BLE and RGE, and D3G on oxaliplatin inhibition. To determine if there was an antagonistic, additive, or synergistic effect, extracts were tested in combination with oxaliplatin. IC_50_ values were utilized: BLE (1.2 mg/mL), RGE (1.4 mg/mL), D3G (395.8 μg/mL), and oxaliplatin (13.5 μg/mL). Extracts (or D3G) were combined with oxaliplatin in a 1:1 ratio of the IC_50_ concentrations as the highest concentration and step-wise dilutions were tested to determine the IC_50_ of the combination. Then, using the expected and observed IC_50,_ the type of effect the extracts and D3G had on oxaliplatin inhibition effectiveness was determined based on the calculated V values^23^.

### Metabolism of phenolics analysis with HPLC and colorimetry

HPLC analysis at 520 and 280 nm of the pure ANC (D3G, C3G, and M3G) was performed in duplicate with pure compounds before application to the cells and in the media following 24 h treatment on HCT 116 cells. A Hitachi HPLC System (Hitachi High Technologies America, Inc., Schaumburg, IL) equipped with a multi-wavelength detector L-7100 pump was utilized with a Prevail C18 column (5 µm, 250 × 4.6 mm, Columbia, MD)^24^.

A Hunter Lab LabScan II (Hunter Associates Laboratory, Inc., Reston, VA) was used to analyze color parameters: L*, a*, and b using CIELAB 10°/D65 for D3G, C3G, and M3G in the media before and after being applied to HCT 116 cells for 24 h in the incubator at 37 °C and 5% CO_2_. This was to confirm the visual color changes seen and offer quantitative data for each ANC treatment. The initial color was compared to the final color of the media that was collected 24 h after treatment on the cells. Calculations were made as described previously^25^.

### Evaluation of apoptosis induction by delphinidin-3-*O*-glucoside and its metabolites using flow cytometry

Since the pure compounds tested were more effective on HCT 116 cells, apoptosis induction was determined for these cells. For apoptosis measurement, HCT 116 cells were seeded at 3 × 10^5^ cells per well in a six-well plate as described before^17^. After 24 h, they were treated with D3G, DC, GA, red grape combination (RGC: D3G, M3G, and Q3G (57:33:10), and oxaliplatin for 24 h at the IC_50_ determined values (ranging from 13.5–395.8 µg/mL). A BD LSR II Flow Cytometry Analyzer (BD Biosciences, San Jose, CA) was used to evaluate the state of 10,000 cells. Duplicate readings of each well were taken, and two independent experiments were analyzed. Total apoptotic cells was a combination of late and early apoptotic cells in quadrants two and four, respectively analyzed through FCS Express 5 Software.

### Immune response analysis

#### Co-culture with PBMC and HCT 116 cells

Day one PBMC were seeded in a 24-well plate at 0.5–1 × 10^6^ cells per mL untreated or with treatments for 48 h: C3G or D3G at 100 μM or pembrolizumab at 6.7 nM. The concentration of 100 μM was used after a pretest (data not shown) showed it had the highest induced cancer cell inhibition even with higher concentrations tested in pretreatment with the PBMC. HCT 116 cells were seeded at 1 × 10^4^ cells per well in a 96 well plate on day two. Day three PBMC from each treatment or untreated cells were centrifuged at 200 g for 15 min. Regular media was added to bring to 1 × 10^6^ cells per mL so that when adding 200 μL to each 96 well of HCT 116 cells, there would be a 10:1 PBMC to HCT 116 cell ratio. After 24 h, viability was determined using a Pierce LDH Cytotoxicity Assay Kit. A visual summary of the method is shown in Supplementary Fig. 1.

#### Effect of pure phenolics and D3G-rich plant extracts on VEGF and PD-L1 protein expression

Western blots for VEGF and PD-L1 were run using a protocol based on that used by Dia and Gonzalez de Mejia^26^. The PD-L1 antibody is a mouse monoclonal IgG_2b_ raised against amino acids 18-238 which represents the partial length of human PD-L1. Briefly, HCT 116 and HT-29 cells were seeded in a six-well plate at 3 × 10^5^ cells per well for 24 h. Then, cells were either left untreated or treated with pure phenolics or the D3G-rich plant extracts (BLE or RGE) at their IC_50_. After another 24 h, cell lysates were obtained to be used in the SDS-PAGE electrophoresis.

#### PD-L1 analysis through ELISA

Human PD-L1 ELISA kit (Abcam ab214565) was used to analyze the effect of the phenolics and extracts in a non-denatured condition with a higher scale of sensitivity. The PD-L1 antibody was rabbit monoclonal with an immunogen of a recombinant full-length protein of human PD-L1 containing the extracellular domain (Phe19-Thr239). The manufacturer’s instructions were followed. Briefly, HCT 116 and HT-29 cells were seeded in a six-well plate at 4 × 10^5^ cells per well for 24 h. Then, cells were left untreated or treated with pure phenolics (D3G, DC, GA, and RGC) or plant extracts (BLE and RGE) at their IC_50_ values. C3G was tested at the same concentration as D3G since it did not have high enough inhibition to have an IC_50_ value. Atezolizumab, a PD-L1 inhibitor drug, was used as a positive control at 6.9 nM (1 μg/mL) as suggested by the manufacturer; ELISA was the only method in which atezolizumab was recommended to be used because of the non-denatured conditions. After another 24 h, cell lysates were obtained by following the Abcam protocol for adherent cells. PD-L1 calculations for samples were based on the standard curve and translated to pg of PD-L1 per μg of protein based on their protein concentration.

#### PD-1 analysis through ELISA in peripheral blood mononuclear cells

Human PD-1 ELISA kit (Invitrogen BMS2214) was used to analyze the effect of C3G and D3G on PD-1 in PBMC in a monoculture and co-culture with HCT 116 and HT-29 cells. PBMC were seeded in a 24-well plate 0.5–2 × 10^6^ cells per mL for 24 h either untreated or treated with C3G or D3G (100 μM). Pembrolizumab, a PD-1 inhibitor drug, was used as a positive control at 6.7 nM (1 μg/mL) as suggested by the manufacturer. After 48 h, the cell culture supernatant was collected post-centrifugation (200 g for 15 min). The media was then centrifuged at 20,817 g (Eppendorf Centrifuge 5417 R, 30 1.5–2 mL tubes) for 10 min at 4 °C and supernatant was collected. To analyze in co-culture with HCT 116 and HT-29 cells, at the step where media was collected post-centrifugation, media was also added to the cell pellet to be added at a 10:1 ratio of PBMC to colorectal cancer cells in a 96-well plate. After 24 h of co-culture, the media was collected, centrifuged, and the supernatant collected as described above. The DC protein assay was used to determine the concentration of protein for all supernatants. For the ELISA, the manufacturer’s instructions were followed. PD-1 calculations for samples were based on the standard curve and translated to pg of PD-1 per μg of protein.

#### PD-L1 analysis through confocal microscopy

Due to higher expression of PD-L1 in HCT 116 cells, further analysis with confocal microscopy was tested in these cells. The PD-L1 antibody was a rabbit monoclonal with an immunogen of a synthetic peptide within amino acids 250 to the C-terminus of human PD-L1. HCT 116 cells were seeded on 8-well immunofluorescence and high-end microscopy slide at 1.5 × 10^4^ cells per well. After 24 h, pure compounds were added, with one untreated well; for D3G and DC, the IC_50_ concentrations were used, and C3G was used at the same concentration as D3G. Following 24 h, cells were washed with PBS and fixed in 4% paraformaldehyde for 30 min. They were permeated with 0.5% Triton X100 after PBS washes for 15 min. Then they were incubated in cold methanol for 15 min at −20 °C and then for 30 min in PBS at room temperature. After 30 min in ITsignal FX, the primary PD-L1 antibody was added at a 1:50 dilution and left overnight at 37 °C. The next day, cells were washed and incubated for 3 h in the secondary antibody at a 1:200 dilution. Cells were washed and drained before adding Prolong Gold. After 24 h in the dark, slides were kept at 4 °C until analyzed using a Zeiss LSM 880 laser-scanning confocal microscope at the Carl R. Woese Institute for Genomic Biology at the University of Illinois. Zen 2 (blue edition) software (Carl Zeiss Microscopy) was used to obtain pictures and measure fluorescence by taking the mean intensity of the PD-L1 signal divided by the mean DAPI signal with the histogram tool.

#### In silico studies of phenolics and immune response related proteins

*In silico* analysis was utilized to screen the potential for phenolic compounds to inhibit immune response related proteins. The 3D structures of PD-1 (5WT9), PD-L1 (5N2D), and VEGF (5FV1) were obtained from Protein Data Bank. Phenolic structures were downloaded from the PubChem Compound database (National Center for Biotechnology Information, Bethesda, MD): C3G (CID: 44256715), PB1 (CID: 11250133), D3G (CID: 443650), M3G (CID: 44257034), DC (CID: 68245), and GA (CID: 370). Water molecules and ligands that accompanied the protein structures were removed in BIOVIA Discover Studio Client 2016. The protein structures were then uploaded to AutoDock Tool where partial Gasteiger charges were automatically implemented and the search space was configured to be a cube with sides measuring 20 angstroms centered on the protein binding sites for each drug and respective ligand. Two sites were tested for each protein with each phenolic ligand: PD-1 at the nivolumab and PD-L1 binding sites; PD-L1 at the atezolizumab and 8J8 small molecule inhibitor site; and VEGF at the VEGFR1 and VEGFR2 binding sites. The search space for the PD-1/PD-L1 binding site was centered to be near VAL64, ILE124, and LEU128 on PD-1^27^. VEGF binding sites to its receptors were estimated to be near ASP63, GLU64, and GLU67 for VEGFR1, and near ARG82, LYS84, and HIS86 for VEGFR2^28^. A related small molecule inhibitor of each protein was used as a comparison. The small molecule 8YZ accompanied by 5NIU was used for PD-1 and PD-L1 sites. For the 8J8 site on PD-L1, the 8J8 molecule that accompanied the 5N2D structure was selected. The control chosen for VEGF at VEGFR1 was vatalanib, obtained from PubChem (CID: 151194). Using MarvinSketch, a structure of PTC-858, a reported VEGF inhibitor, was replicated from ChemSpider (CID: 4501829)^29^. Protein structures were transferred to AutoDock Tools as rigid molecules. Each phenolic and small molecule inhibitor was also uploaded to AutoDock Tools as a ligand and their number of flexible bonds was reduced to half their maximum torsions. After these alterations, all molecules were saved as PDBQT files before docking experiments were run. The data received from AutoDock Vina was analyzed in BIOVIA Discovery Studio 2016 Client to determine the nature of each docking interaction and 3D configuration.

#### Fluorescence quenching of anthocyanins and immune checkpoint proteins

To confirm docking predictions seen with C3G and D3G and immune checkpoints, fluorescence spectroscopy analysis was used to determine whether an interaction occurs and if it is static or dynamic. A FluoroMax-3 spectrofluorometer (HORIBA Jobin Yvon, Edison, NJ) was used with C3G and D3G and recombinant human PD-L1 protein and human PD-1 protein combinations. The PD-L1 protein was kept constant at 10 nM and PD-1 at 100 fM in distilled deionized water. Excitation wavelength was set to 280 nm, increment at 2 nm, integration time of 0.5 s, excitation and emission slits at 5 nm, scan start at 310 nm, and scan end at 500 nm. The peak at 354 nm was determined to be the main peak of the PD-L1 and PD-1 proteins. Concentrations of anthocyanins (1000, 500, 250, 100, and 10 µM) were incubated with the fixed concentrations of the proteins to read fluorescence intensity. The Stern-Volmer equation system was utilized as described previously^30^. The lifetime of the fluorophore of protein in the absence of a quencher, τ0, used was 10^−8^.

### Statistical analysis

Statistical analyses were conducted using one-way ANOVA to compare experimental to control values with JMP version 8.0. Comparisons between groups for viability and apoptosis were performed using the Tukey- Kramer test; differences were considered significant at p < 0.05. For western blots and confocal microscopy, comparisons between the untreated control and treatments were performed using Student’s test; differences were considered significant at p < 0.05. At least two independent experiments run with at least duplicate data points were performed for every study.

## Results

### HCT 116 and HT-29 cell viability inhibition by delphinidin-3-*O*-glucoside and its metabolites

**Figure 1:**
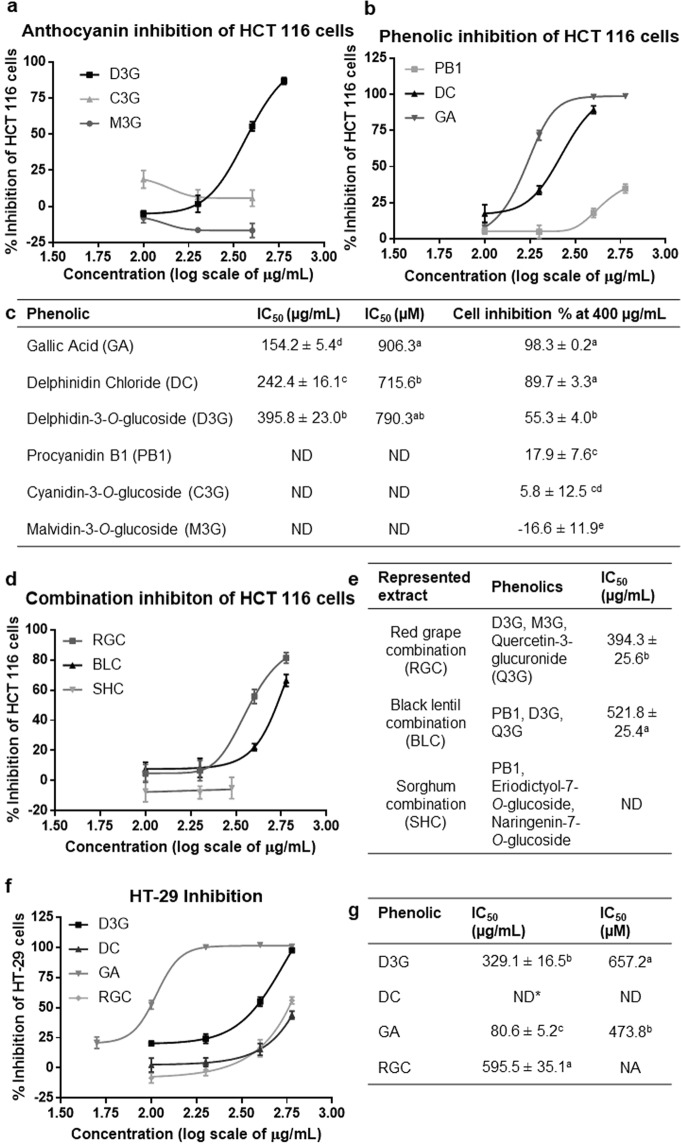
Inhibition curves with standard error bars. (**a**) Pure anthocyanins on HCT 116 cells. (**b**) Pure phenolics on HCT 116 cells. (**c**) The 50% viability inhibition concentrations (IC_50_) of the pure anthocyanins and phenolics on HCT 116 cells (mean ± SEM) and their percent inhibition compared at 400 μg/mL. (**d**) Inhibition curves of the pure phenolic combinations on HCT 116 cells. (**e**) The IC_50_ values of the pure phenolic combinations on HCT 116 cells (mean ± SEM). (**f**) Inhibition curves of the pure phenolics and red grape combination on HT-29 cells. (**g**) The IC_50_ values of the pure phenolics and red grape combination on HT-29 cells (mean ± SEM). Letters indicate statistical difference between phenolics per column as determined by the Tukey test (p < 0.05). Table c presents the statistics for the IC_50_ inhibitions of HCT 116 cells by phenolics and anthocyanins. Table (**e**) presents the statistics for the IC_50_ inhibitions of HCT 116 cells by combination of phenolics and anthocyanins present in represented extracts. Table (**g**) presents the statistics for the IC_50_ inhibitions of HT-29 cells by phenolics and anthocyanins; statistics are compared within the table only for each column. At least two independent experiments were run with triplicate data points. *Inhibition for delphinidin chloride at 600 μg/mL was 43.9%. GA, gallic acid; DC, delphinidin chloride; D3G, delphinidin-3-*O*-glucoside; PB1, procyanidin B1; C3G, cyanidin-3-*O*-glucoside; M3G, malvidin-3-*O*-glucoside; RG, red grape; BL, black lentil; SH, sorghum; NA, not applicable; ND, not able to be determined.

Of the three ANC (D3G, C3G, and M3G) and PB1 tested on HCT 116 cells, only D3G showed a dose-response inhibitory effect with an IC_50_ at 395.8 μg/mL. Figure 1 shows the inhibition curves, IC_50_’s, and inhibition at 400 μg/mL of the pure phenolics for comparison. Both of the D3G metabolites tested, DC and GA, inhibited HCT 116 cells with IC_50_ of 242.4 and 154.2 μg/mL for DC and GA, respectively (Fig. 1a–c). For the phenolic combinations (Fig. 1d,e), pure compounds from red grape had the highest potency with an IC_50_ at 394.3 μg/mL, not statistically different from D3G alone at 395.8 μg/mL (p = 0.9671). Since the RGC was the most potent, it was also tested on HT-29 cells and had an IC_50_ of 589.9 μg/mL, statistically higher (p = 0.0021) than that for HCT 116 cells. D3G and GA had IC_50_ for HT-29 cells of 329.1 and 80.6 μg/mL, respectively; DC had up to 43.5% inhibition at 600 μg/mL (Fig. 1f,g). The IC_50_ for D3G on HT-29 cells was not statistically different from its IC_50_ for HCT 116 cells (p = 0.0608).

### Black lentil and red grape extracts had an additive effect on oxaliplatin inhibition of HCT 116 cells, but delphinidin-3-*O*-glucoside had a slightly antagonistic effect

**Figure 2:**
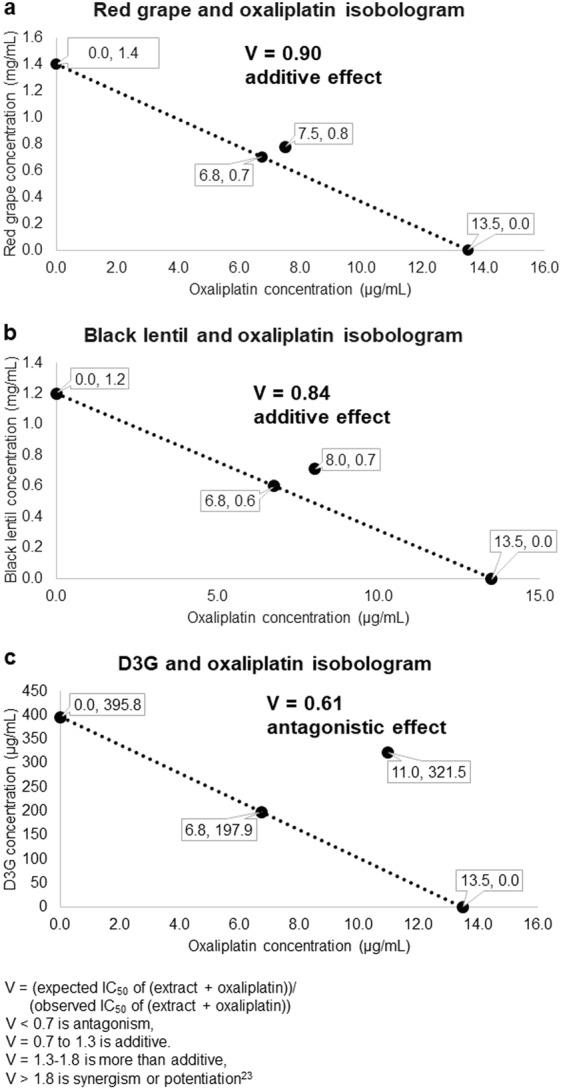
Isobolograms of anthocyanin-rich extracts or delphinidin-3-*O*-glucoside and oxaliplatin with their V values and the type of effect based on the V value ranges. (**a**) Red grape. (**b**) Black lentil. (**c**) Delphinidin-3-*O*-glucoside. D3G, delphinidin-3-*O*-glucoside. Two independent experiments were performed with triplicate data points.

Figure 2 shows that both BLE and RGE had an additive effect with oxaliplatin. The V values were 0.84 and 0.90 for BLE and RGE respectively, putting them in the additive range (0.7–1.3)^23^. However, D3G had a V value of 0.61, which just put it in the antagonistic range (V < 0.7).

### Transformation of anthocyanins that inhibited cell viability after incubation with HCT 116 cells

When performing the cell viability tests with ANC, notable color changes in the culture media occurred after 24 h of treatment on HCT 116 cells as shown in Supplementary Fig. 2. D3G had a more drastic color change (Supplementary Fig. 2a), while C3G and M3G (Supplementary Fig. 2b,c) had dulling of the color, but the media appeared to better retain its hue angle. Supplementary Fig. 2 shows that D3G is no longer present (at 520 nm) in media, after the 24 h treatment on the cells, as there was no peak at the D3G retention time detectable by the HPLC software. However, for C3G and M3G the major peak remains after 24 h treatment, at lower peak intensity. Colorimeter tests in Supplementary Table 1 show a similar decrease in saturation, dulling of the color, for all three ANC but a larger change in L*, a*, and b* parameters for D3G.

### Delphinidin-3-*O*-glucoside and its metabolites induced apoptosis in HCT 116 cells

**Figure 3:**
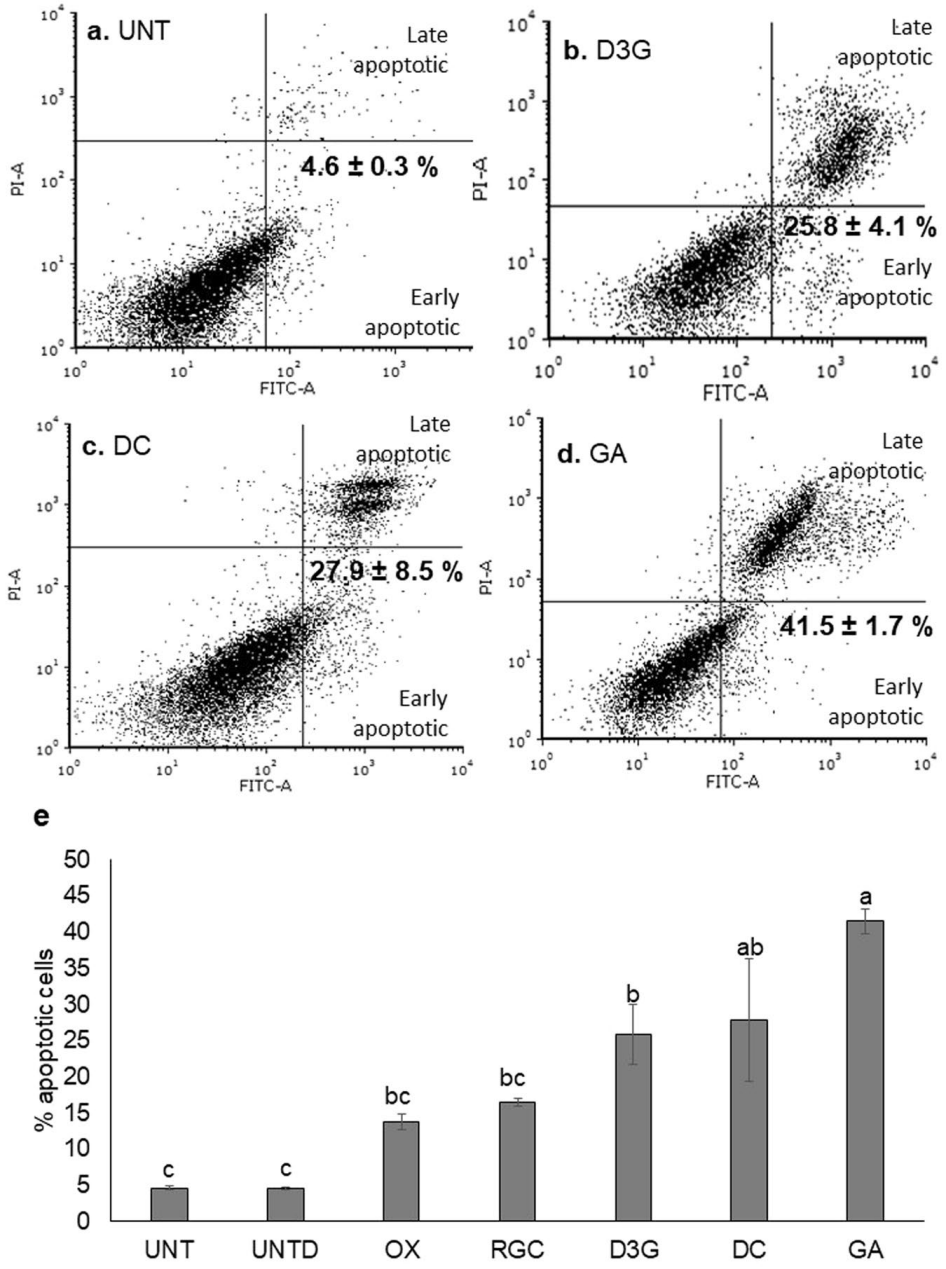
Flow cytometry plots (propidium iodide (PI) vs annexin V FITC (fluorescein isothiocyanate) and percentages of total apoptotic HCT 116 human colon cancer cells. The total of apoptotic cells is the combined percentage of early and late apototic cells, quadrants two and four, respectively. (**a**) Untreated. (**b**) Delphinidin-3-*O-*glucoside. (**c**) Delphinidin chloride. (**d**) Gallic acid. (**e**) Percent of apoptotic cells for all tested treatments. The results are expressed as mean ± SEM. Different letters indicate statistical difference among all treatments as determined by Tukey test p < 0.05. At least two independent experiments with duplicate data points were performed. D3G, delphinidin-3-*O-*glucoside; DC, delphinidin chloride; OX, oxaliplatin; RGC, red grape combination; UNT, untreated; UNT D, untreated DMSO. RGC was compared to UNT D; both had 0.3% DMSO.

Figure 3 shows the results of apoptosis induction by D3G, its metabolites (DC and GA), and the pure compound combination RGC, in comparison to untreated cells. There was a significant increase in apoptotic cells due to D3G, DC, and GA (p values of 0.0075, 0.0035, and <0.0001, respectively). GA had the highest percentage of apoptotic cells (41.5%), followed by DC (27.9%) and D3G (25.8%). The increase of apoptotic cells caused by RGC was not statistically higher than the untreated DMSO control (p = 0.1735). The basis for the quadrant placements is shown in Supplementary Fig. 3.

### Cyanidin-3-*O*-glucoside treatment of peripheral blood mononuclear cells prior to co-culture with HT-29 cells increased LDH activity

Supplementary Fig. 4 shows the LDH activity results for the co-culture. Compared to the untreated HCT 116 monoculture, LDH activity increased for each co-culture of PBMC with HCT 116 cells by 53.3% for the untreated, 47.0% for the D3G pretreated, 49.5% for the C3G pretreated, and 74.5% for the pembrolizumab pretreated (p values of 0.0114, 0.0242, 0.0303, and 0.0034, respectively) (Supplementary Fig. 4a). There was no statistical difference between the untreated PBMC and the pre-incubation of PBMC with C3G, D3G, or pembrolizumab (p values of 0.3254, 0.9229, and 0.9799, respectively). Compared to the untreated HT-29 monoculture, LDH activity increased for each co-culture of PBMC with HT-29 cells by 51.1% for the untreated, 61.0% for the D3G pretreated, 93.8% for the C3G pretreated, and 118.0% for the pembrolizumab pretreated (p values of 0.0141, 0.0101, 0.0009, and 0.0002, respectively). There was a significant increase induced by C3G and pembrolizumab compared to the untreated co-culture (p values of 0.0489 and 0.0098, respectively) (Supplementary Fig. 4b).

### Delphinidin-3-*O*-glucoside, its metabolites, and D3G-rich plant extracts decreased expression of VEGF and PD-L1

**Figure 4:**
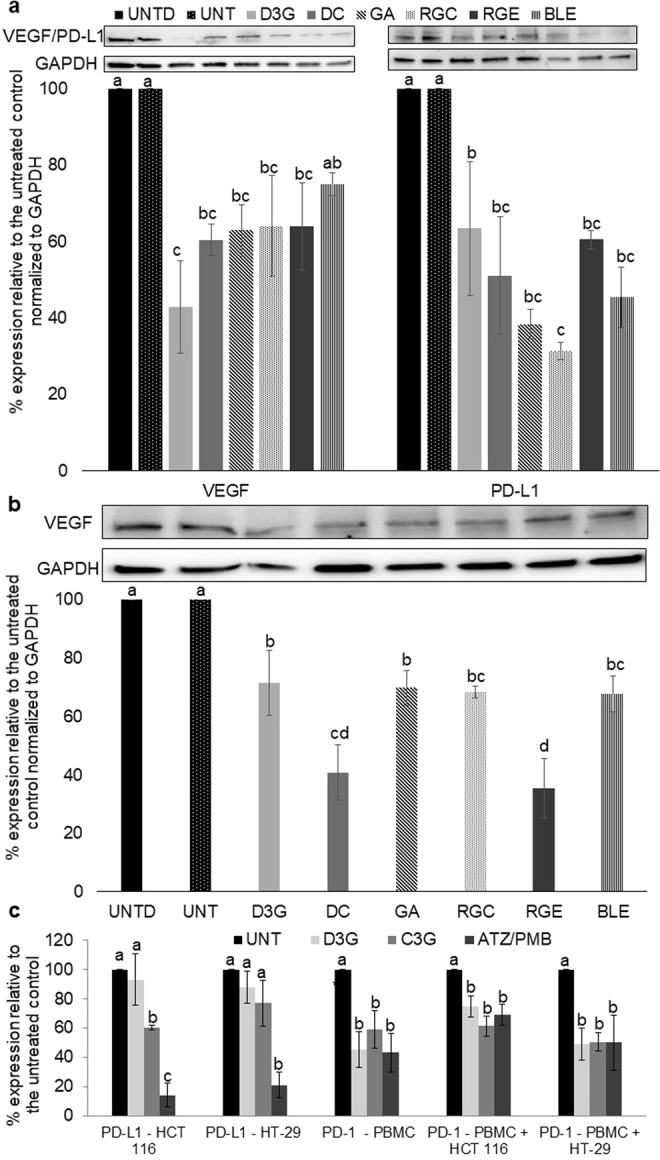
Comparison of the percent of protein expression relative to the untreated (UNT) control normalized to GAPDH for vascular endothelial growth factor (VEGF) and programmed death-ligand 1 (PD-L1) for delphinidin-3-O-glucoside (D3G), delphinidin chloride (DC), red grape combination (RGC), red grape extract (RGE), and black lentil extract (BLE) and the associated protein band examples for (**a**) HCT 116 human colon cancer cells. (**b**) HT-29 human colon cancer cells (VEGF only). RGC was compared to untreated DMSO (UNTD); both had 0.3% DMSO. Western blot pictures were cropped and full-length blots are shown in Supplementary Figs 6 and 7. The results are expressed as mean ± standard error. Different letters indicate statistical difference between treatments and the untreated control (100%) as determined by Student’s T-test (p < 0.05) per protein tested per cell line. At least two independent experiments were run. c. ELISA results for PD-L1 and programmed cell death protein 1 (PD-1) in terms of percent inhibition compared to the untreated control in monoculture or co-culture. The results are expressed as mean ± standard error. Asterisks indicates statistical difference from the untreated control as determined by Student’s T-test (p < 0.05) compared within each monoculture or coculture tested. GAPDH, glyceraldehyde 3-phosphate; MW, molecular weight; C3G, cyandin-3-O-glucoside; PBMC, peripheral blood mononuclear cells; ATZ, atezolizumab; PMB, pembromolizumab.

Figure 4 shows the western blot results for D3G, DC, GA, RGC, RGE, and BLE with VEGF for both HCT 116 and HT-29 cells. In HCT 116 cells (Fig. 4a), all treatments decreased VEGF expression significantly compared to the untreated cells except BLE, with D3G decreasing the protein expression 57.2%. In HCT 116 cells, all treatments decreased the expression of PD-L1 compared to the untreated control, with RGC reducing the protein expression by 68.7%. C3G did not significantly decrease the PD-L1 expression with 20.7% inhibition compared to the untreated control (p > 0.05) (data not shown in the figure). In HT-29 cells (Fig. 4b), all treatments reduced VEGF expression compared to the control, with RGE inducing the largest decrease (64.6%) and not statistically different than DC (59.1%). PD-L1 expression was too low in HT-29 cells to evaluate the effect of the treatments through western blots since well-developed bands could not be elucidated.

### Cyanidin-3-*O*-glucoside was the only non-pharmaceutical treatment to inhibit PD-L1 expression through ELISA analysis

Results are shown for D3G, C3G, and atezolizumab in Fig. 4c.

In both HCT 116 and HT-29 cells, there were no significant inhibitions of PD-L1 by any of the D3G related pure compounds or extracts. However, C3G significantly inhibited PD-L1 in HCT 116 cells at 39.5% (p = 0.0110). Atezolizumab had high inhibition of PD-L1 in both cell lines: 85.9% for HCT 116 and 79.1% for HT-29 cells compared to the untreated control cells. It was also shown that the expression of PD-L1 was much higher in pg per µg of total protein in HCT 116 cells vs. HT-29 cells with 3.7 times the amount of PD-L1 protein in HCT 116 cells.

### Cyanidin-3-*O*-glucoside and delphinidin-3-*O*-glucoside inhibit PD-1 through ELISA analysis in both monoculture and co-culture

C3G and D3G decreased PD-1 expression in peripheral blood mononuclear cells in a monoculture by 41.0% and 54.8% (p = 0.0339 and 0.0152, respectively), and in a co-culture with HCT 116 and HT-29 cells by 38.7% and 25.6% (p = 0.0007 and 0.0093, respectively), and 49.5% and 51.0% (p = 0.0378 and 0.0345, respectively), respectively, compared to the untreated control as show in Fig. 4c.

### Cyanidin-3-*O*-glucoside decreased fluorescence intensity of PD-L1 in HCT 116 cells

**Figure 5:**
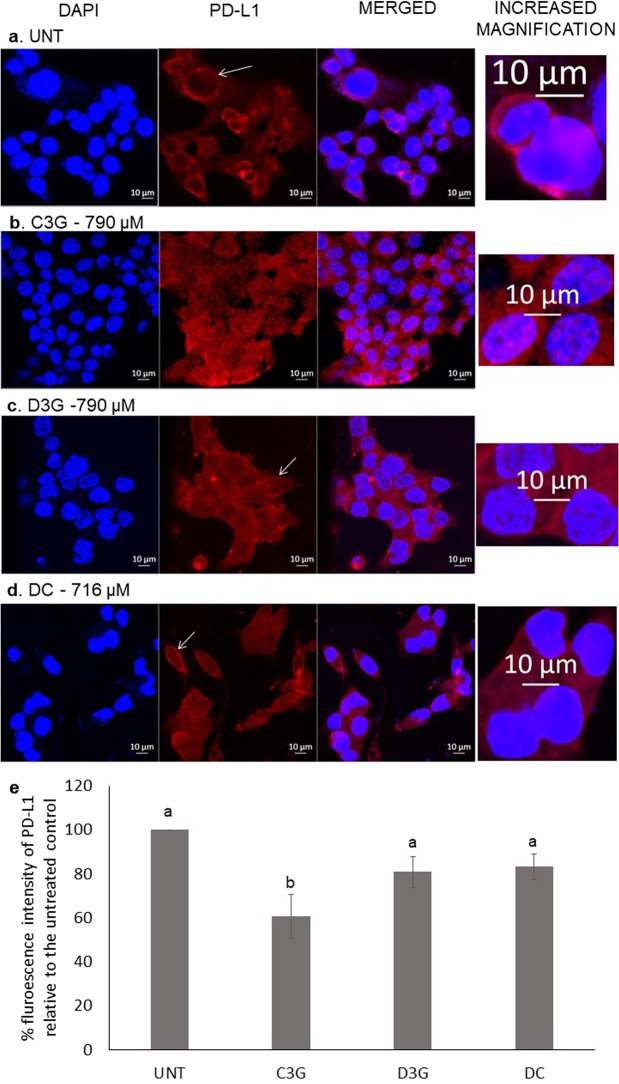
Effect of anthocyanins and delphindin chloride (DC) on programmed death-ligand 1 (PD-L1) localization in the cell and fluorescence intensity. Examples of confocal microscopy pictures of cells. (**a**) Untreated (UNT), (**b**) Cyanidin-3-*O*-glucoside (C3G), (**c**) Delphinidin-3-*O*-glucoside (D3G). (**d**) DC. (**e**) The relative fluorescence intensity of PD-L1 for treatments of C3G, D3G, and DC compared to the untreated control. The results are expressed as mean ± SEM. Different letters indicate statistical difference between treatments and the untreated control (100%) as determined by Student’s T-test (p < 0.05). At least two independent experiments were run. At least three sections of a well were used to quantify the expression of PD-L1 in Zen 2 (blue edition) software. White arrows indicate visualization of membrane localization of the protein.

Figure 5 shows examples of microscopy pictures from untreated HCT 116 cells and C3G, D3G, and DC treated cells revealing more intense fluorescence of PD-L1 in the untreated cells. C3G, D3G, and DC decreased intensity compared to the control by 39.2%, 19.1%, and 16.7%, respectively (p values of 0.0346, 0.2455, and 0.3193, respectively). Visually, there appears to be higher intensity of the PD-L1in the membrane for the untreated HCT 116 cells.

### Delphinidin-3-*O*-glucoside and cyanidin-3-*O*-glucoside show potential to inhibit VEGF and immune checkpoints *in silico*

**Figure 6:**
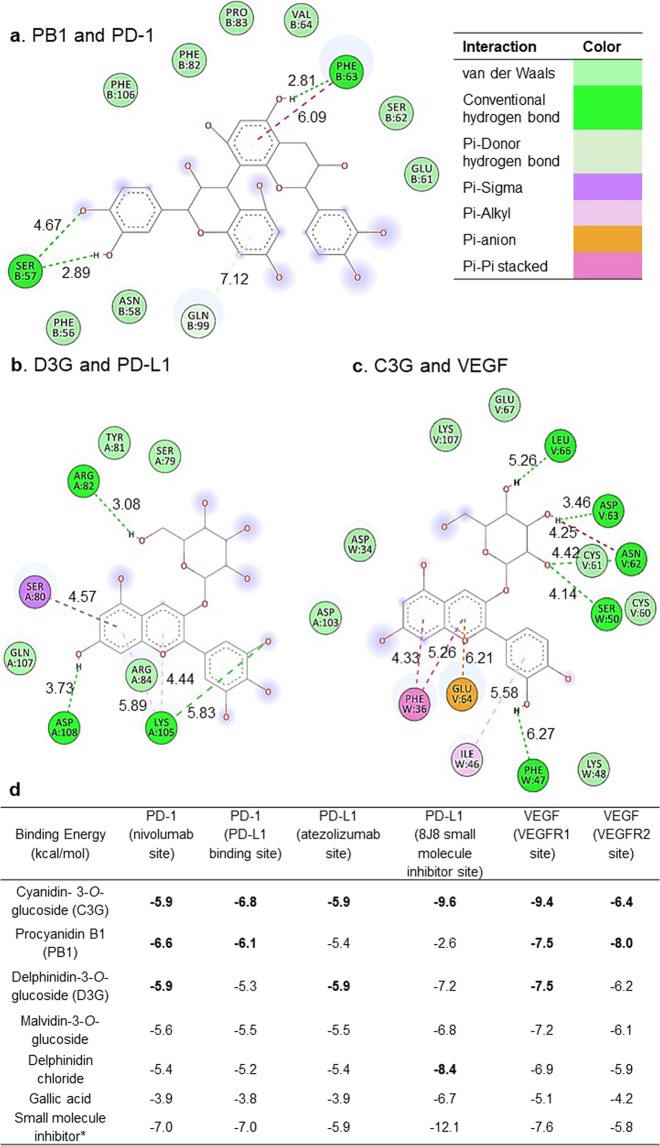
Two-dimensional figures including distances of interactions between an immune checkpoint or vascular endothelial growth factor (VEGF) protein and a phenolic ligand for (**a**) Procyanidin B1(PB1) and programmed cell death protein 1 (PD-1). (**b**) Delphinidin-3-*O*-glucoside (D3G) and programmed death-ligand 1(PD-L1). (**c**) Cyanidin-3-*O*-glucoside (C3G) and VEGF. (**d**) Free energy of binding for two sites on PD-1, PD-L1, or VEGF with phenolic ligands: C3G, PB1, D3G, malvidin-3-*O*-glucoside, delphinidin chloride, gallic acid, and a small molecule inhibitor. The top two or three phenolics with the highest binding affinities are bolded. *The small molecule 8YZ was used for PD-1 and PD-L1 sites except for the 8J8 site. For the 8J8 site on PD-L1, the actual 8J8 molecule was selected. Vatalanib was utilized for VEGF at VEGFR1 and PTC-858 for VEGFR2. Lengths given in the figures are in angstroms.

All tested phenolics (D3G, C3G, DC, M3G, GA, and PB1) were predicted to bind to immune checkpoints (PD-1 and PD-L1) and VEGF (Fig. 6). For the drug binding sites of PD-1 and PD-L1, C3G and D3G (−5.9 kcal/mol) had the highest binding affinity. For PD-1 at the binding site of PD-L1, C3G and PB1 had a binding affinity of −6.8 and −6.1 kcal/mol, respectively. For PD-L1 at a small molecule inhibitor site, C3G and DC had the most negative predicted free energy of binding with −9.6 and −8.4 kcal/mol, respectively. For VEGF, C3G, PB1, and D3G had the highest binding affinity for both the VEGFR1 and VEGFR2 binding sites. Common interactions between C3G and the small molecule inhibitor for PD-L1 were a polar interaction with SER93 and a hydrophobic interaction with LEU50 (Supplementary Table 4). D3G shared a polar interaction with GLN99 with the small molecule inhibitor for PD-1. The immune checkpoint CTLA4 was also modeled with the phenolics at the site of the drug ipilimumab (data not shown). D3G and C3G also had highest binding affinity of the phenolics, with −5.6 and −5.3 kcal/mol, respectively; GA had the lowest at −3.6 kcal/mol. The two-dimensional modeling pictures of the ANC ligands in the immune checkpoint proteins and VEGF are shown in Fig. 6 and the three-dimensional pictures in Supplementary Fig. 5. There were several similar amino acid interactions not only among the phenolics but also among the phenolics and the small molecule inhibitor used as a comparison for each protein. These interactions are shown in Supplementary Tables 2, 3 and 4 for PD-1, PD-L1, and VEGF, respectively.

### Delphinidin-3-*O*-glucoside and cyanidin-3-*O*-glucoside demonstrate static quenching of immune checkpoints

**Figure 7:**
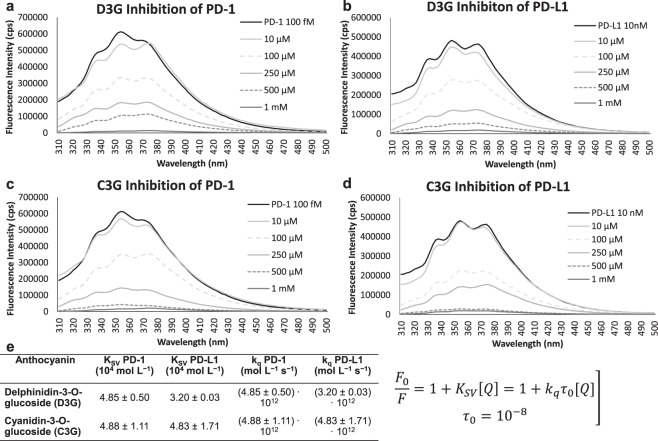
Fluorescence quenching of immune checkpoints programmed cell death protein 1 (PD-1) and programmed death-ligand 1 (PD-L1) by anthocyanins. Representative fluorescence spectra for (**a**) Delphinidin-3-*O*-glucoside (D3G) with PD-1 (**b**) D3G with PD-L1 (**c**) Cyanidin-3-*O*-glucoside (C3G) and PD-1 and (**d**) C3G and PD-L1. (**e**) Table showing K_sv_ and k_q_ values given as the mean ± standard error of mean, with two independent replicates of each concentration (1 mM, 500 µM, 250 µM, 100 µM, 10 µM). No statistical difference among K_sv_ values (p > 0.05) for each protein and anthocyanin interaction as determined by Tukey-Kramer. (**f**) The Stern-Volmer equation where F_0_ is the fluorescence intensity of the protein peak at 354 nm without a quencher, F is the fluorescence intensity at a given concentration of quencher [Q], K_sv_ is theh Stern–Volmer quenching constant, k_q_ is the bimolecular quenching constant, and 𝜏_0_ is the average lifetime of protein without quencher, value as defined in the figure.

Figure 7 shows the fluorescence spectra of anthocyanins, D3G and C3G, with immune checkpoints, PD-1 and PD-L1. F_0_/F was plotted against concentration to obtain the K_sv_ and then k_q_ values. The calculated k_q_ values for each interaction demonstrate a static quenching indicating a formation of a complex between the immune checkpoint proteins and anthocyanins based on the k_q_ values being higher (320–488 times) than the diffusion-limited quenching in water of 10^10^ mol^−1^ s^−1^ ^30^.

## Discussion

Recent *in vitro* and *in vivo* studies have reported the inhibitory effects of ANC-rich extracts from a wide range of sources on proliferation and progression of colon cancer cells; however, little has been shown on ANC-rich extracts on the immune response in the tumor microenvironment^31–34^. Additionally, a lesser number of studies have analyzed the ability of pure ANC to inhibit colorectal cancer progression *in vitro*^35,36^. We discovered that the only ANC tested that was effective at colorectal cancer cell inhibition in HCT 116 cells was D3G. Another study found that M3G was not effective, but metabolites, GA, 3-*O*-methylgallic acid, and 2,4,6-trihydroxybenzaldehyde were successful inhibiting HCT 116 cells, as well as other colorectal cancer cells^37^. In our study, we found that GA had a dose-dependent inhibition in both cell lines, HCT 116 and HT-29. It was expected that combining ANC and phenolics in ratios representative of phenolic-rich extracts would result in synergistic effects on the inhibition of HCT 116 cells. However, this was not seen in either cell line as determined by the RGC not being statistically better than D3G in HCT 116 cells; in HT-29 cells, D3G was significantly more effective than RGC. A study testing berry anthocyanidins (aglycones of ANC) on lung cancer cell inhibition found delphinidin as the only anthocyanidin that did not have a significantly higher inhibition with a mixture of phenolics compared to single anthocyanidin^38^. In the animal portion of the study, the mixture and delphinidin both exerted antitumor activity that was not statistically different. Delphinidin was the only anthocyanidin that was effective inhibiting the two lung cancer cell lines tested alone, similar to the trend in our study where D3G was the only effective ANC. D3G and its metabolites appear to play a key role in the anti-carcinogenic activity of the plant extracts.

It was demonstrated that the ANC-rich extracts did not negatively affect the ability of oxaliplatin to decrease colorectal cancer viability; however, D3G did have a slightly antagonistic effect. In a study looking at the effects of the phenolic compound quercetin on cisplatin (also in the platinum-containing family of drugs) in ovarian cancer models, it was concluded that quercetin had an antagonistic effect on cisplatin at low concentrations^39^. However, quercetin and its related glycoside, rutin, have been reported to decrease peripheral neuropathy, a common side effect of oxaliplatin treatment in mice that received biweekly injections of oxaliplatin^40^.

Significant color changes and lack of HPLC peak retention coincided with greater efficacy of D3G to inhibit HCT 116 viability in comparison to C3G and M3G. These observations led us to believe that cell uptake and transformation of D3G was occurring in the HCT 116 cells, the metabolites of D3G were having an impact on viability, or a combination of both. Comparing the structure of C3G versus D3G, the difference is that D3G has an additional hydroxyl group on its B-ring. More hydroxyl groups have been connected to less stable pigment but also to higher antioxidant capacity^41^. A study using Caco-2 cells testing black currant anthocyanins found that delphinidins were both less stable and had higher initial absorption efficiency than the cyanidin derivatives^42^. These studies corroborate our findings of a more considerable color change and reduction of cancer cell viability induced by D3G versus C3G. Additionally, with a higher degradation of D3G, it is likely that the degradation products of D3G were also impacting the reduction in cell viability.

We found that D3G, DC, and GA induced apoptosis significantly, providing further mechanistic knowledge. A study that analyzed grape and strawberry ANC-rich extracts found that the extracts and one of the metabolites induced apoptosis^43^. Additionally, a java plum extract containing glucosides of delphinidin was shown to increase apoptosis in HCT 116 cells^44^. These studies provide evidence that ANC-rich extracts can stimulate apoptosis, including those that have D3G or its derivatives as a main component of the ANC profile. Here, we looked at individual components and metabolites and showed that D3G alone can induce apoptosis, as well as its metabolites, DC and GA. Analysis continued in relation to angiogenesis and immune response by determining VEGF expression effects. In both HCT-116 and HT-29 cells, VEGF expression decreased due to D3G, DC, GA, and D3G-rich treatments (RGC, BLE, and RGE). A study discovered that pomegranate juice extract that contained 3-glucosides and 3,5-diglucosides of delphinidin, cyanidin, and pelargonidin was successful at reducing VEGF expression in HT-29 cells^45^. In the *in silico* analysis, we found that C3G, PB1, and D3G had the most potential to inhibit VEGF at the VEGFR1 and VEGFR2 sites of the protein, but ANC and metabolites may have more of an affinity at the VEGFR1 site. Previously, we demonstrated that D3G and C3G had a high binding affinity with VEGFR2^18^. Also, research on ellagic acid, a phenolic compound abundant in plant foods, showed anti-angiogenic potential through docking analysis by revealing strong bonds at the ATP site of the VEGFR-2 kinase domain^46^. We build on the *in silico* angiogenesis and immune response analysis to show that these ANC also have a potential to inhibit VEGF, a ligand of VEGFR1 and VEGFR2.

Immunotherapy techniques have been identified as pivotal strategies to implement in combination with other therapies, to combat cancer progression and resistance^47^. D3G, DC, GA, and D3G-rich treatments (RGC, BLE, and RGE) reduced PD-L1 protein expression compared to the untreated HCT 116 cells. Based on the docking results, C3G was shown to have the highest potential of all the pure compounds to inhibit PD-L1 at the small inhibitor site. At the sites of the drug inhibitors for both PD-1 and PD-L1, C3G and D3G had the same predicted binding energy. This was confirmed in fluorescence quenching analysis, where C3G and D3G showed static quenching with both PD-1 and PD-L1 with no statistical difference in Stern-Volmer constants. These results confirmed the potential of C3G and D3G to form complexes with immune checkpoint proteins. C3G was able to inhibit PD-L1 expression in the ELISA and as shown through lowering fluorescence intensity of PD-L1 where D3G and its metabolites did not. Despite higher expression of PD-L1 in HCT 116 cells and the microsatellite stability differences (HCT 116 instable and HT-29 stable), atezolizumab was successful at inhibiting the expression in both cell lines. D3G, its related metabolites and extracts, and C3G inhibited PD-L1, which could decrease the binding of PD-L1 to PD-1 resulting in activation of T cells in the tumor microenvironment. D3G and C3G also inhibited PD-1 expression not only in PBMC cells in a monoculture but as pretreatments in a co-culture model with both HCT 116 and HT-29 cells; this showed their potential to block both immune suppressive proteins. Additionally, C3G pre-treated PBMC increased cytotoxicity of HT-29 cells in a co-culture. An *in vivo* hepatocellular cancer study with *Lonicera caerulea* ‘Beilei’ fruit showed this ANC-rich extract was able to alter the immunoregulatory activity^48^. ANC-rich black raspberries were researched in an *in vivo* esophageal cancer model and were reported to positively modulate cytokine and immune cell response, focusing on the innate immune response^49^. These studies showed a potential of ANC-rich extracts to promote an immune response in other cancers. A recent *in vivo* study using aronia berry demonstrated the ability of ANC-rich extracts to hinder colitis progression through T cell modulation^50^. Here, we add to the potential mechanisms of how ANC and ANC-rich extracts may affect the immune response, specifically demonstrating their impact on immune checkpoints.

**Figure 8:**
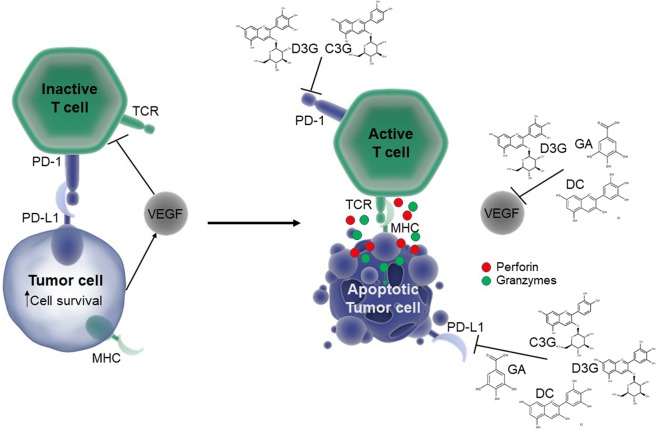
Summary of the effects of anthocyanins and metabolites on the immune response markers tested. Inhibition (bar-headed lines) or promotion (arrows) shown if effects were seen through western blots, ELISA, confocal microscopy, and/or *in silico* analysis. C3G, cyanidin-3-*O*-glucoside; D3G, delphinidin-3-*O*-glucoside; DC, delphinidin chloride; GA, gallic acid; MHC, major histocompatibility complex; PD-1, programmed cell death protein 1; PD-L1, programmed death-ligand 1; TCR, T cell receptor; VEGF, vascular endothelial growth factor.

In summary, this study showed that D3G and its metabolites, DC and GA, inhibited HCT 116 and HT-29 human colon cancer cell viability in a dose-dependent manner and induced apoptosis. D3G treatment on HCT 116 cells, produced significant changes in chromatographic profile and color parameters in cell media correlating with its inhibition ability. *In silico* screening showed potential for ANC and their metabolites to inhibit immune checkpoints and VEGF, with C3G indicating overall more favorable binding energies. Despite C3G not reducing the viability of either colorectal cancer cell line directly, PBMC pre-treated with C3G increased cytotoxicity of HT-29 cells, and C3G inhibited PD-L1 *in vitro*. D3G, its metabolites, and D3G-rich treatments (RGC, BLE, and RGE) decreased the expression of PD-L1 and VEGF proteins. C3G and D3G inhibited PD-1 expression in both a monoculture of PBMC, as well as through pre-incubation of PBMC in a co-culture with HCT 116 and HT-29 cells. A summary of the pure phenolic effects seen in this study are shown in Fig. 8. The results of this research demonstrated the potential of ANC to promote immune destruction of colorectal cancer cells by inhibiting negative regulators, immune checkpoints, of the adaptive response in both colorectal cancer cells and immune cells. To our knowledge this research shows for the first time, the potential for pure ANC and their metabolites to inhibit immune checkpoints. This contributes to the understanding of the anti-carcinogenic potential of ANC-rich foods, opening further exploration of their stimulatory effects on the immune response.

## Supplementary information

Supplementary Figures and Tables

## Data Availability

Availability of data and material: All data generated or analyzed during this study are included in this published article [and its supplementary information files].
