# Supplementary material for: Anthocyanins, delphinidin-3-O-glucoside and cyanidin-3-O-glucoside, inhibit immune checkpoints in human colorectal cancer cells in vitro and in silico
Source: Sci Rep. 2019 Aug 9;9:11560. doi: 10.1038/s41598-019-47903-0 (PMC6689002; doi:10.1038/s41598-019-47903-0)
Supplement: Supplementary file 1 — Supplementary Figures and Tables [file 41598_2019_47903_MOESM1_ESM.pdf]

**Anthocyanins, delphinidin-3-*O*-glucoside and cyanidin-3-*O*-glucoside, inhibit immune checkpoints in human colorectal cancer cells *in vitro* and *in silico***

Candice Mazewski: cmazews2@illlinois.edu

Sanha Kim: msk2@illinois.edu

Elvira Gonzalez de Mejia: edemejia@illinois.edu

## Supplementary Figure Legends

**Supplementary Figure 1**. Summary of the method used for co-culture of peripheral blood mononuclear cells (PBMC) and colorectal cancer cells for viability and supernatant collection for programmed cell death protein 1 (PD-1) ELISA analysis. HCT 116 cells are shown as an example of colorectal cancer cells in the figure; the same procedure was used for HT-29 cells. UNT, untreated; C3G, cyanidin-3-O-glucoside; D3G, delphinidin-3-O-glucoside; PMB, pembrolizumab; LDH, lactate dehydrogenase.

**Supplementary Figure 2.** High-performance liquid chromatography profiles and picture of a well from a 96-well plate of phenolics in media at 520 and 280 nm initially and after incubation with HCT 116 human colon cancer cells at 37 ˚C in 5% CO2 and 95% air. a. Delphinidin-3-O-glucoside (D3G). b. Cyanidin3-O-glucoside (C3G). c. Malvidin3-O-glucoside (M3G). Two independent experiments were run.

**Supplementary Figure 3.** Flow cytometry cell count versus apoptosis staining pictures to show where quadrants were place in Figure 3. Red line indicates the quadrant placement which is positioned just before the second cluster of cells to include the higher intensity stained cells for Annexin V fluorescein isothiocyanate (FITC) and propidium iodide (PI). a. Untreated b. Delphinidin-3-O-glucoside (D3G) c. Delphinidin chloride (DC) d. Gallic Acid.

**Supplementary Figure 4**. Lactate dehydrogenase (LDH) activity of co-cultures with untreated and anthocyanin pre-treated peripheral blood mononuclear cells (PBMC) and colon cancer cells compared to the untreated monoculture of colon cancer cells. a. HCT 116 cells. b. HT-29 cells. UNT, untreated; C3G, cyanidin-3-O-glucoside; D3G, delphinidin-3-O-glucoside; PMB, pembrolizumab.

**Supplementary Figure 5.** Three-dimensional figures and binding free energy between a. Cyanidin-3-O-glucoside (C3G) and programmed cell death protein 1 (PD-1) at the programmed death-ligand 1 (PD-L1) binding site. b. Delphinidin-3-O-glucoside (D3G) and PD-1 at the nivolumab binding site. c. Delphinidin (DC) and programmed death-ligand 1 (PD-L1) at the 8J8 small molecule inhibitor site. d. Malvidin-3-O-glucoside (M3G) and PD-L1 at the Atezolizumab binding site. e. Procyanidin B1 (PB1) and vascular endothelial growth factor (VEGF) at the VEGFR2 binding site. f. Gallic acid (GA) and VEGF at the VEGFR1 binding site.

**Supplementary Figure 6.** Full-length uncropped western blot images depicting a. programmed death-ligand 1 PD-L1 for HCT 116 cells b. glyceraldehyde 3-phosphate (GAPDH) for HCT 116 cells for the PD-L1 membrane c. vascular endothelial growth factor (VEGF) for HCT 116 cells d. GAPDH for HCT 116 cells for the VEGF membrane for the cropped figures in Fig. 4a. e. VEGF for HT-29 cells f. GAPDH for HT-29 cells for VEGF membrane for cropped figures in Fig. 4b. MCF7 whole cell lysate (sc-2206) was used in lane 10 as a control for the VEGF membranes as recommended by the manufacturer. Recombinant human PD-L1 protein fragment (ab167713) was used in lane 10 of the PD-L1 membrane. BLE, black lentil extract; D3G, delphinidin-3-O-glucoside; DC, delphinidin chloride; GA, gallic acid; RGC, red grape combination; RGE, red grape extract; UNT, untreated; UNTD, untreated DMSO.

**Supplementary Figure 7.** Full-length uncropped western blot images depicting additional exposures as adjusted in Carestream for Supplementary Fig. 6a for programmed death-ligand 1 PD-L1 for HCT 116 cells a. increased exposure b. decreased exposure. For Supplementary Fig. 6c for vascular endothelial growth factor (VEGF) for HCT 116 cells c. increased exposure d. decreased exposure. For Supplementary Fig. 6e for VEGF for HT-29 cells e. increased exposure f. decreased exposure. MCF7 whole cell lysate (sc-2206) was used in lane 10 as a control for the VEGF membranes as recommended by the manufacturer. Recombinant human PD-L1 protein fragment (ab167713) was used in lane 10 of the PD-L1 membrane. BLE, black lentil extract; D3G, delphinidin-3-O-glucoside; DC, delphinidin chloride; GA, gallic acid; RGC, red grape combination; RGE, red grape extract; UNT, untreated; UNTD, untreated DMSO.

Supplementary Figure 1

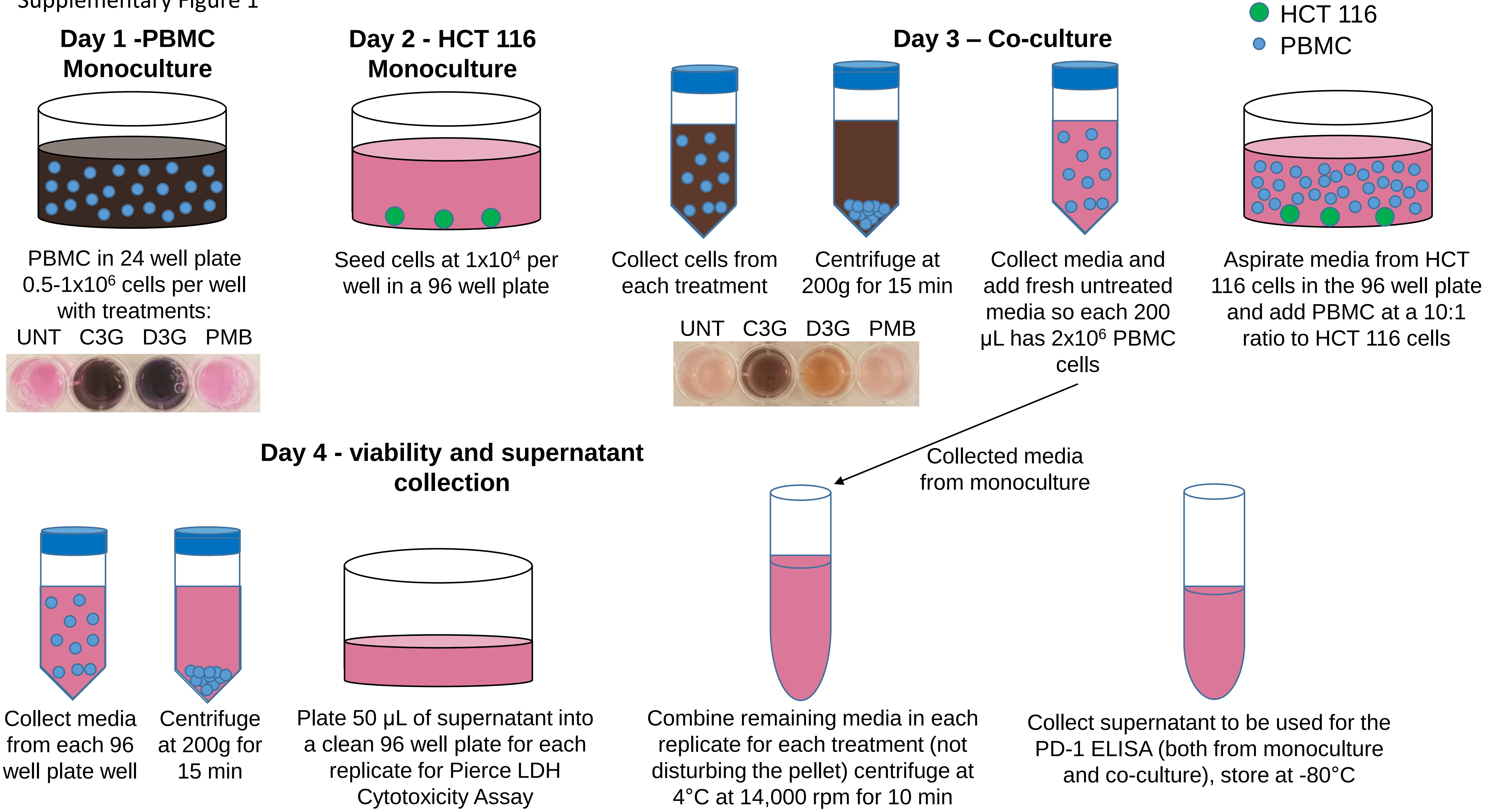

Supplementary Figure 2

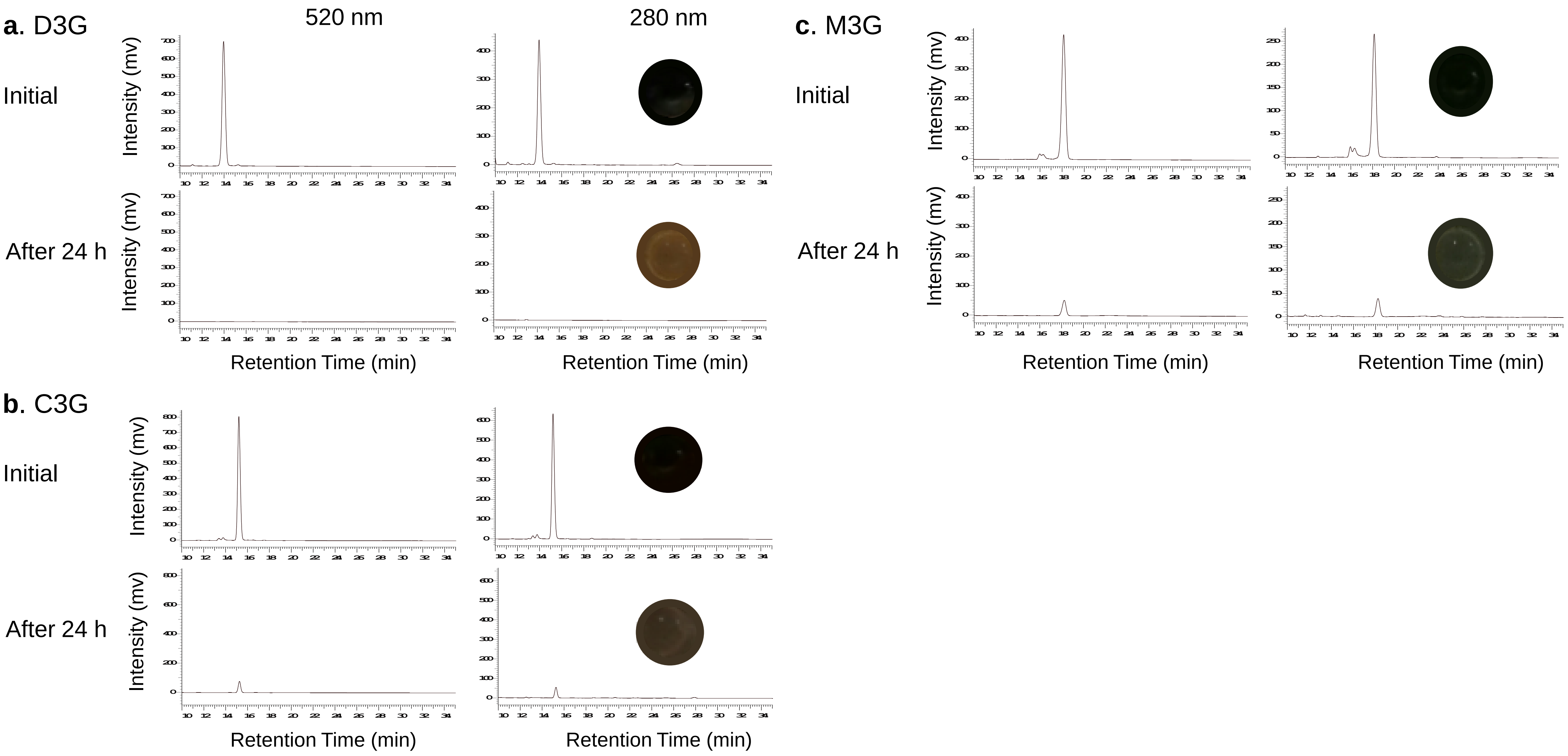

Supplementary Figure 3

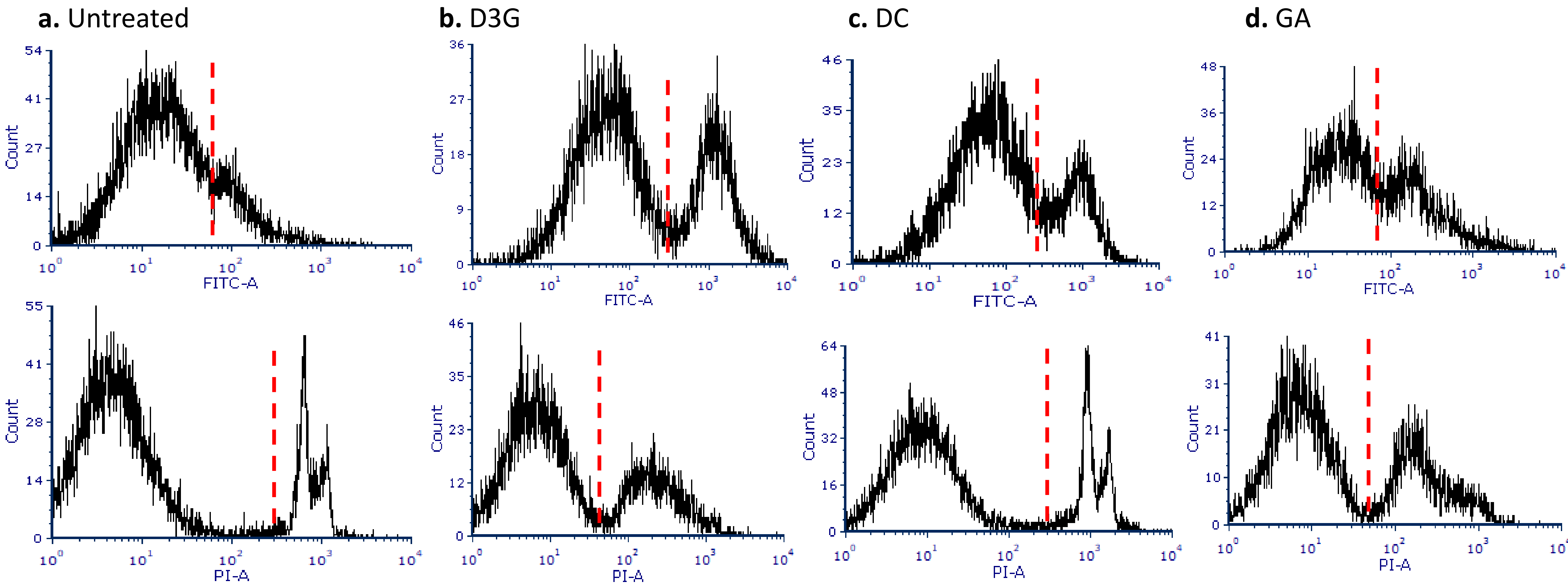

Supplementary Figure 4

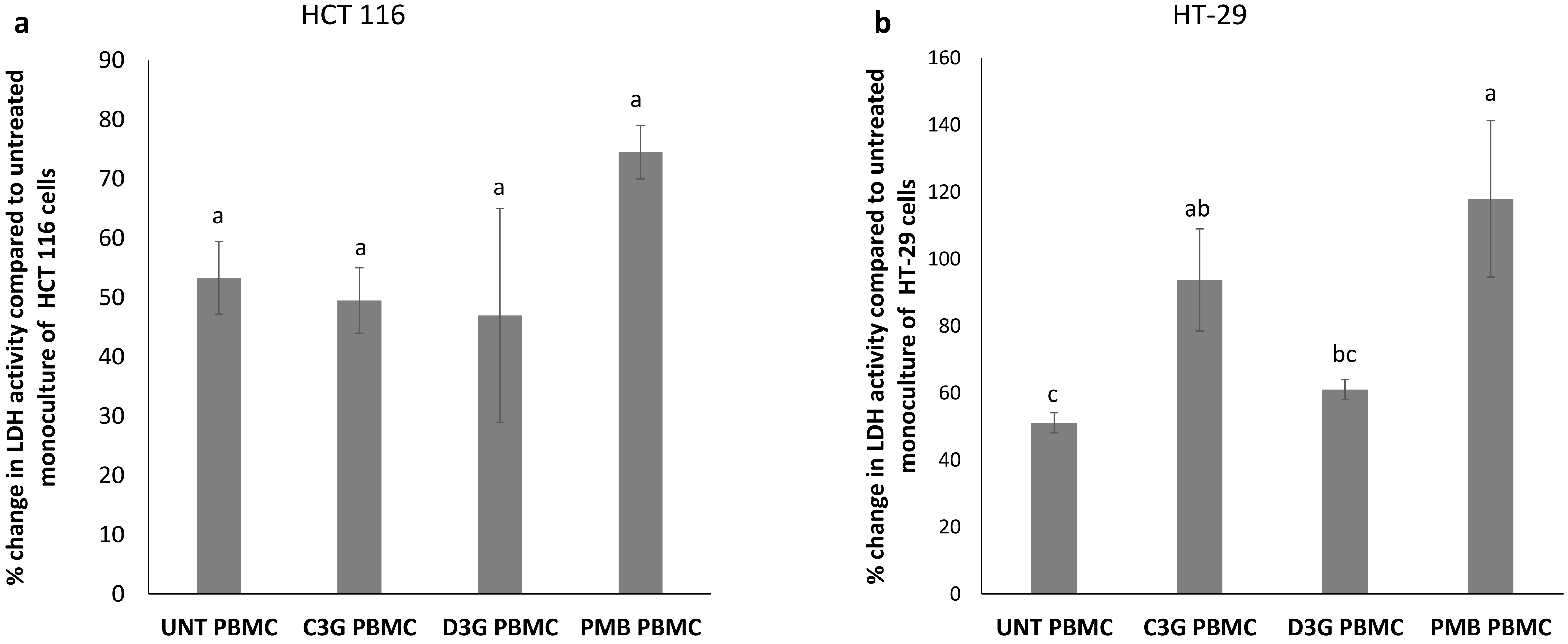

Supplementary Figure 5

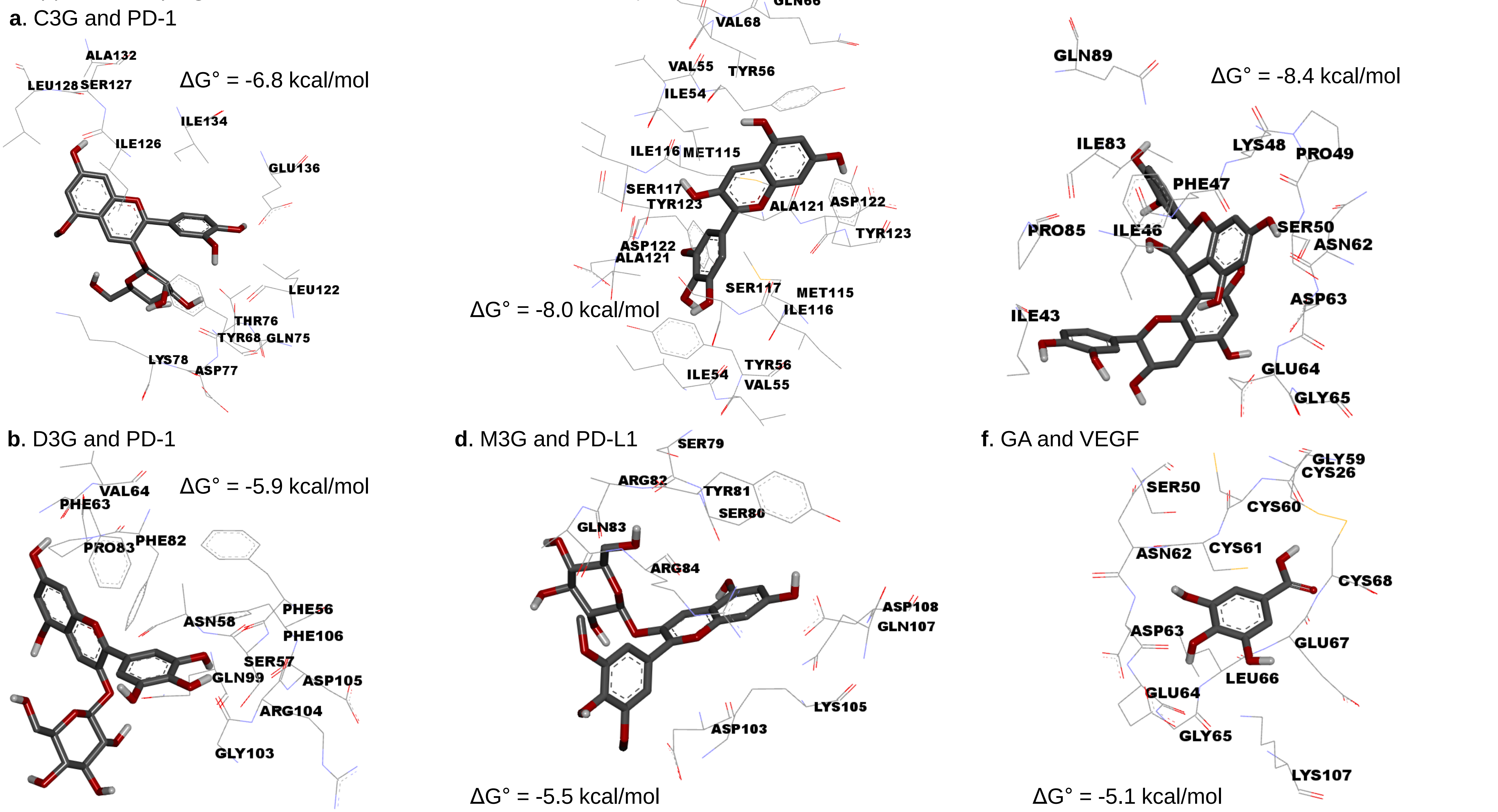

Supplementary Figure 6

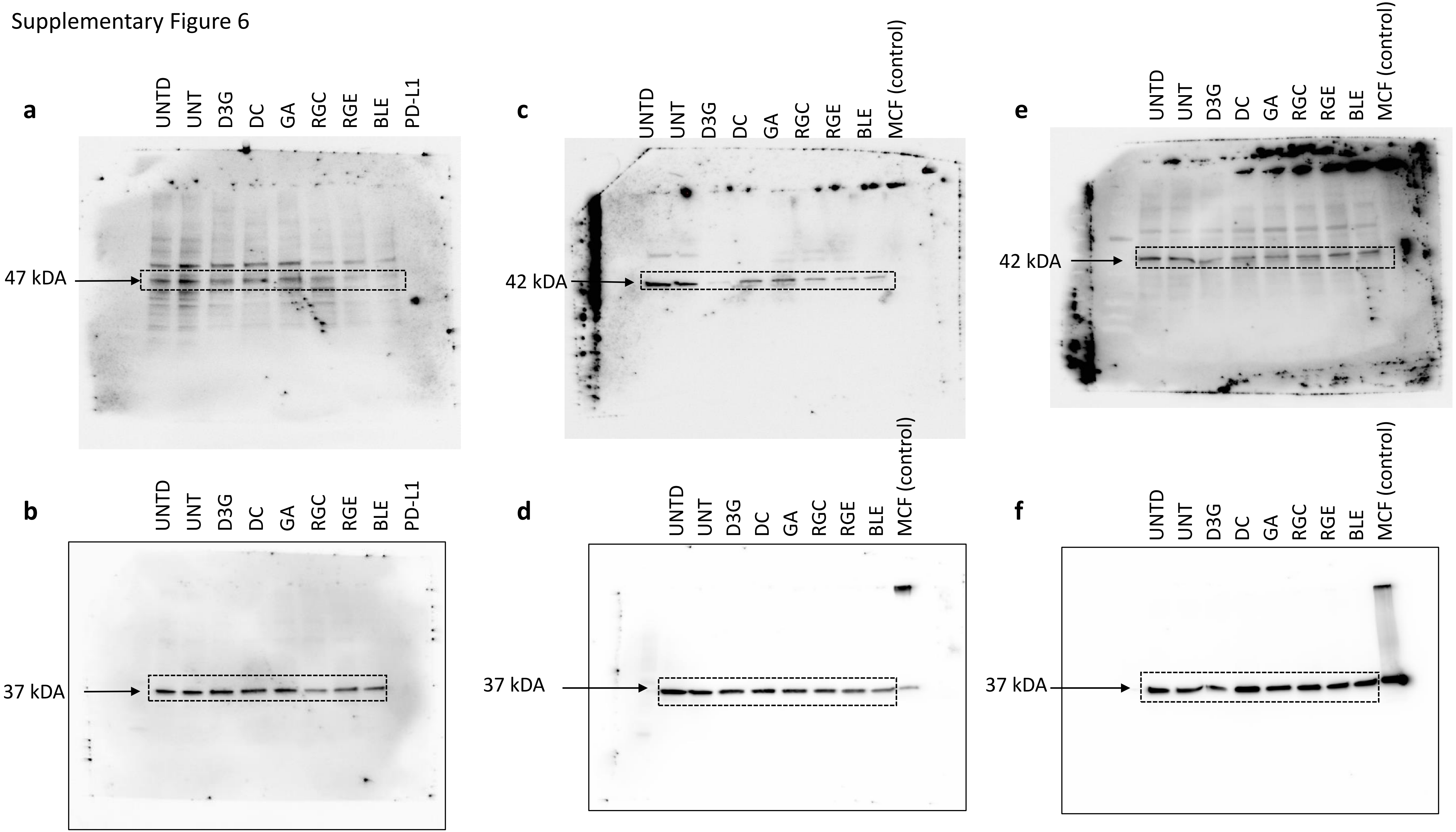

Supplementary Figure 7

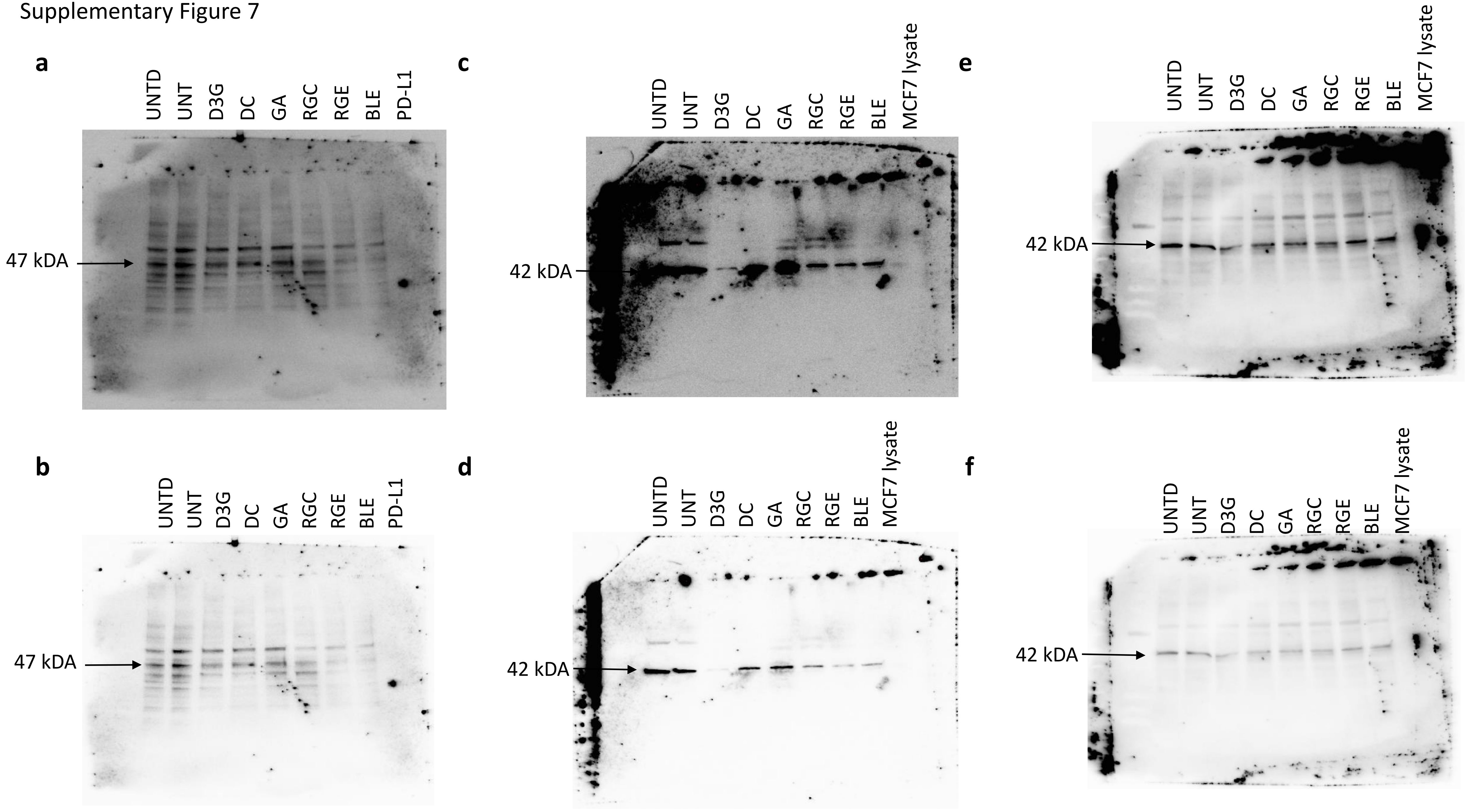

**Supplementary Table 1:** Comparison of color parameters of anthocyanins in the media initially and after incubation with HCT 116 cells for 24 h at 37˚C.

| | | L* | a* | b* | Hue Angle | Chroma | Saturation | ΔE | Color |
|---|---|---|---|---|---|---|---|---|---|
| Cyanidin-3-*O*-Glucoside | Initial | 1.3 ± 0.1 | 9.8 ± 0.6 | 0.9 ± 0.7 | 5.1 ± 4.5 | 9.8 ± 0.6 | 7.6 ± 0.1 | | |
| | After 24 h | 10.9 ± 1.3 | 23.7 ± 1.8 | 13.3 ± 1.3 | 29.2 ± 1.3 | 27.1 ± 1.8 | 2.5 ± 1.3 | | |
| | **Δ** | 9.6 | 13.9 | 12.4 | 24.2 | 17.3 | -5.1 | 20.9 | |
| Delphinidin-3-*O*-Glucoside | Initial | 0.3 ± 0.0 | 1.8 ± 0.2 | 0.3 ± 0.1 | 10.4 ± 5.2 | 1.8 ± 0.2 | 6.5 ± 0.8 | | |
| | After 24 h | 28.6 ± 2.5 | 19.7 ± 0.3 | 40.0 ± 1.3 | 63.7 ± 1.0 | 44.6 ± 1.1 | 1.6 ± 0.1 | | |
| | **Δ** | **28.4** | **18.0** | **39.7** | **53.3** | **42.9** | -5.0 | **52.0** | |
| Malvidin-3-*O*-Glucoside | Initial | 0.3 ± 0.0 | 1.9 ± 0.0 | 0.4 ± 0.0 | 13.2 ± 0.1 | 1.9 ± 0.0 | 7.5 ± 0.1 | | |
| | After 24 h | 8.7 ± 2.1 | 3.7 ± 1.4 | 7.9 ± 0.7 | 64.9 ± 8.0 | 8.7 ± 0.8 | 1.0 ± 0.2 | | |
| | **Δ** | 8.4 | 1.8 | 7.4 | 51.8 | 6.8 | **-6.5** | 11.4 | |

All parameters were statistically different ($p < 0.05$) between the initial and after 24 h per anthocyanin except for a* for malvidin-3-*O*-glucoside.

**Supplementary Table 2:** Estimated free energy of binding and interactions between amino acids of the phenolic ligand (or small molecule inhibitor) and protein, programmed cell death protein 1 (PD-1).

| Ligand | PD-1 Binding Site | Binding Energy (kcal/mol) | Hydrogen Bonding | van der Waal Forces | π-Interactions | Carbon-Hydrogen Bonds |
|---|---|---|---|---|---|---|
| **Cyanidin-3-*O*-glucoside** | Nivolumab | -5.9 | PHE56, **SER57**, SER62, PHE63 | THR59, SER60, **GLU61**, **PHE82**, PRO83, **GLN99**, **ARG104**, PHE106 | | ASN58, GLY103 |
| | PD-L1 | -6.8 | TYR68, GLN75, THR76 | LYS78, LEU122, LEU128, ALA132 | **ILE126**, **ILE134** | |
| **Procyanidin B1** | Nivolumab | -6.6 | **SER57**, PHE63 | PHE56, **ASN58**, **GLU61**, **SER62**, **VAL64**, **PHE82**, PRO83, PHE106 | GLN99 | |
| | PD-L1 | -6.1 | LYS78, GLY124, GLU136 | VAL64, ASN66, THR76, LEU122, ALA125, LEU128 | **ILE126**, **ILE134** | TYR68 |
| **Delphinidin-3-*O*-glucoside** | Nivolumab | -5.9 | PHE56, **SER57**, PHE63, GLN99, GLY103 | **ASN58**, **VAL64**, **ARG104**, PHE106 | PHE82, **PRO83** | |
| | PD-L1 | -5.3 | | ASN66, LYS78, LEU122, GLY124, ALA132, ILE134 | **ILE126** | TYR68, THR76 |
| **Malvidin-3-*O*-glucoside** | Nivolumab | -5.6 | GLU61 | PHE56, SER57, **ASN58**, SER60, **SER62**, PHE63, **PHE82**, **GLN99**, GLY103, **ARG104**, PHE106 | | |
| | PD-L1 | -5.5 | ASN66, THR76, LYS78 | VAL64, GLY124, ALA132, **GLN133**, ILE134, GLU136 | **ILE126** | TYR68 |
| **Delphinidin chloride** | Nivolumab | -5.4 | PHE56, **SER57**, PHE63 | **ASN58**, **VAL64**, GLY103, **ARG104**, PHE106 | **PHE63**, PHE82, **PRO83** | PRO83 |
| | PD-L1 | -5.2 | THR76, GLU136 | LEU122, GLY124, LEU128, ALA132 | TYR68, **ILE126**, **ILE134** | |
| **Gallic acid** | Nivolumab | -3.9 | ASN33, **SER57**, SER60, SER127, GLN133 | PHE56, **ASN58**, THR59, LYS135 | | |
| | PD-L1 | -3.8 | GLU136 | **TYR68**, THR76, LEU122, GLY124, ILE126 | **ILE134** | |
| **8YZ (small molecule inhibitor)** | Nivolumab | -7.0 | **SER57** | **ASN58**, **GLU61**, **SER62**, **VAL64**, **PHE82**, **GLN99**, PRO101, ASN102, **ARG104** | PHE56, **PHE63**, **PRO83**, GLY103, PHE106 | |
| | PD-L1 | -7.0 | ILE134 | **TYR68**, PRO83, **GLN133** | VAL64, **ILE126**, LEU128, ALA132, **ILE134** | |

Bolded interactions are those that are the same between the phenolics and the small molecule inhibitor; Underlined interactions are those that are the same between phenolics.

**Supplementary Table 3:** Estimated free energy of binding and interactions between amino acids of the phenolic ligand (or small molecule inhibitor) and protein, programmed death-ligand 1(PD-L1).

| Ligand | PD-L1 Binding Site | Binding Energy (kcal/mol) | Hydrogen Bonding | van der Waal Forces | π-Interactions | Carbon-Hydrogen Bonds |
|---|---|---|---|---|---|---|
| **Cyanidin-3-*O*-glucoside** | Atezolizumab | -5.9 | | SER79, **SER80**, **ASP103**, **GLN107**, ASP108 | **ARG82**, **LYS105** | |
| | 8J8 small molecule | -9.6 | **TYR56**, ARG125 | ALA18, PHE19, **ILE54**, ASP61, ASN63, GLN66, **VAL68**, **VAL76**, MET115, **LYS124** | **TYR56**, **ALA121**, **ASP122**, **TYR123** | THR20, **ASP122** |
| **Procyanidin B1** | Atezolizumab | -5.4 | ARG82, THR102 | SER79, **SER80**, GLN83, **ASP103**, **GLN107** | **ARG82**, **ARG84**, **LYS105** | |
| | 8J8 small molecule | -2.6 | ASP61, ASN63, TYR123 | THR20, TYR56, LYS62, GLN66, LYS75, HIS78, SER79, **ASP122** | VAL76, **TYR123**, LYS124, ARG125 | |
| **Delphinidin-3-*O*-glucoside** | Atezolizumab | -5.3 | SER79, ASP103 | TYR81, ARG82, **GLN107**, ASP108 | SER80 | ARG84 |
| | 8J8 small molecule | -5.9 | PHE19, THR20, **TYR56**, ASP61, LYS124, ARG125 | ALA18, GLU58, LYS62, ASN63, **TYR123** | VAL76, **ASP122**, LYS124 | |
| **Malvidin-3-*O*-glucoside** | Atezolizumab | -5.5 | SER79, ARG82, ARG84 | TYR81, GLN83, **GLN107**, ASP108 | SER80, ASP103, **LYS105** | |
| | 8J8 small molecule | -6.8 | THR20, GLN66, LYS124 | PHE19, VAL21, TYR56, GLU58, LYS62, ASN63, **TYR123**, **ARG125** | **ASP122**, LYS124 | |
| **Delphinidin chloride** | Atezolizumab | -5.4 | ASP103, ASP108 | ARG82, GLN83, **GLN107** | SER80, **ARG82**, **LYS105** | |
| | 8J8 small molecule | -8.4 | SER117* | **ILE54***, TYR56*, GLN66, **ILE116***, **SER117***, **ASP122***, **TYR123*** | **TYR56***, **MET115***, **ALA121*** | |
| **Gallic acid** | Atezolizumab | -3.9 | ARG84 | **SER80**, ASP108 | **LYS105** | |
| | 8J8 small molecule | -6.7 | ILE54, MET115* | VAL55, TYR56, MET115*, **ILE116**, ALA121*, **ASP122***, **TYR123** | **MET115***, **ALA121*** | SER117 |
| **8YZ (small molecule inhibitor)** | Atezolizumab | -5.9 | GLN83 | LYS62, **SER80**, THR102, **ASP103**, **GLN107** | **ARG82**, **ARG84**, **LYS105** | |
| **8J8 (small molecule inhibitor)** | 8J8 small molecule | -12.1 | **TYR56*** | **ILE54***, **VAL68**, **VAL76**, **ILE116***, **SER117***, **ASP122***, **TYR123***, **LYS124**, **ARG125** | **TYR56***, **MET115***, **ALA121***, **ASP122***, **TYR123*** | GLN66, **ASP122***, TYR123* |

Bolded interactions are those that are the same between the phenolics and the small molecule inhibitor; Underlined interactions are those that are the same between phenolics
*The 5N2D PD-L1 protein structure used is a homodimer and for these ligands identical amino acids from both PD-L1 protein chains participated in binding interactions.

**Supplementary Table 4:** Estimated free energy of binding and interactions between amino acids of the phenolic ligand (or small molecule inhibitor) and protein, vascular endothelial growth factor (VEGF).

| Ligand | VEGF Binding Site | Binding Energy (kcal/mol) | Hydrogen Bonding | van der Waal Forces | $\pi$-Interactions | Carbon-Hydrogen Bonds |
|---|---|---|---|---|---|---|
| **Cyanidin-3-*O*-glucoside** | VEGFR1 | -9.4 | PHE47, **SER50**, ASN62, **ASP63**, LEU66 | **ASP34**, **LYS48**, **CYS60**, **CYS61**, GLU67, LYS107 | PHE36, **ILE46**, **GLU64** | |
| | VEGFR2 | -6.4 | SER50, GLU64 | **PHE47**, PRO49, ASN62, **ASP63**, PRO85, HIS86, **GLN89** | **ILE46**, LYS48, **ILE83** | |
| **Procyanidin B1** | VEGFR1 | -7.5 | GLY59, GLU64, CYS68 | CYS26, **ASP34**, **PHE36**, ILE46, PHE47, SER50, **ASN62**, ASP63, LEU66, GLU67 | CYS60, CYS61, **GLU64**, LYS107 | |
| | VEGFR2 | -8.0 | **ASN62**, ASP63 | **PHE47**, PRO49, **SER50**, GLY65, **GLN89** | **ILE46**, LYS48, GLU64, **ILE83**, PRO85 | |
| **Delphinidin-3-*O*-glucoside** | VEGFR1 | -7.5 | **SER50**, CYS68 | **ASP34**, **PHE36**, ILE46, **LYS48**, **GLY59**, **ASN62**, ASP63, LEU66, GLU67, **ILE83** | CYS60 | CYS61 |
| | VEGFR2 | -6.2 | PHE47, GLN89 | PRO49, **SER50**, ASN62, **ASP63**, GLU64, PRO85 | **ILE46**, LYS48, **ILE83** | |
| **Malvidin-3-*O*-glucoside** | VEGFR1 | -7.2 | ASP34, **ASP63** | PHE47, **LYS48**, SER50, CYS51, **CYS60**, **CYS61**, **ASN62**, LEU66, GLU67, LYS107 | PHE36, **ILE46**, **GLU64** | |
| | VEGFR2 | -6.1 | **ASN62**, GLN89 | **PHE47**, PRO49, **SER50**, **ASP63**, GLU64 | **ILE46**, LYS48, **ILE83** | PRO85 |
| **Delphinidin chloride** | VEGFR1 | -6.9 | **SER50**, **ASP63** | **ASP34**, **PHE36**, **LYS48**, **CYS60**, LEU66, **CYS68**, **ILE83** | **ILE46**, CYS61, ASP63, **GLU64** | GLU67 |
| | VEGFR2 | -5.9 | SER50, **ASN62** | **PHE47**, PRO49, **ASP63**, GLU64, PRO85, **GLN89** | **ILE46**, LYS48, **ILE83** | SER50 |
| **Gallic acid** | VEGFR1 | -5.1 | GLY59, CYS61, ASN62, **ASP63**, LYS107 | CYS26, SER50, **CYS60**, GLU64, LEU66, GLU67, **CYS68** | | |
| | VEGFR2 | -4.2 | SER50, **ASN62** | **PHE47**, PRO49, **ASP63**, GLU64, ILE83 | **ILE46**, LYS48 | SER50 |
| **Vatalanib** | VEGFR1 | -7.6 | **SER50**, **ASP63** | **ASP34**, **PHE36**, **LYS48**, **GLY59**, **CYS60**, **CYS61**, **ASN62**, **CYS68**, **ILE83** | **ILE46**, **GLU64** | PHE47 |
| **PTC-858 (small molecule inhibitor)** | VEGFR2 | -5.8 | **ASN62** | **PHE47**, LYS48, **SER50**, **ASP63**, **GLN89** | **ILE46**, ASN62, **ILE83** | GLU64 |

Bolded interactions are those that are the same between the phenolics and the small molecule inhibitor; Underlined interactions are those that are the same between phenolics.
